# Kappa chain maturation helps drive rapid development of an infant HIV-1 broadly neutralizing antibody lineage

**DOI:** 10.1038/s41467-019-09481-7

**Published:** 2019-05-16

**Authors:** Cassandra A. Simonich, Laura Doepker, Duncan Ralph, James A. Williams, Amrit Dhar, Zak Yaffe, Lauren Gentles, Christopher T. Small, Brian Oliver, Vladimir Vigdorovich, Vidya Mangala Prasad, Ruth Nduati, D. Noah Sather, Kelly K. Lee, Frederick A. Matsen IV, Julie Overbaugh

**Affiliations:** 1 Division of Human Biology, Fred Hutchinson Cancer Research Center, Seattle, WA 98109 USA; 2 Medical Scientist Training Program, University of Washington School of Medicine, Seattle, WA 98195 USA; 3 Public Health Sciences Division, Fred Hutchinson Cancer Research Center, Seattle, WA 98109 USA; 4 Department of Medicinal Chemistry, University of Washington, Seattle, WA 98195 USA; 5 Department of Statistics, University of Washington, Seattle, WA 98195 USA; 6 Department of Microbiology, University of Washington, Seattle, WA 98195 USA; 7 Division of Basic Sciences, Fred Hutchinson Cancer Research Center, Seattle, WA 98109 USA; 8 Center for Infectious Disease Research, Seattle, WA 98109 USA; 9 Department of Pediatrics and Child Health, University of Nairobi, Nairobi, Kenya

**Keywords:** Antibodies, Antibodies, HIV infections

## Abstract

HIV-infected infants develop broadly neutralizing plasma responses with more rapid kinetics than adults, suggesting the ontogeny of infant responses could better inform a path to achievable vaccine targets. Here we reconstruct the developmental lineage of BF520.1, an infant-derived HIV-specific broadly neutralizing antibody (bnAb), using computational methods developed specifically for this purpose. We find that the BF520.1 inferred naive precursor binds HIV Env. We also show that heterologous cross-clade neutralizing activity evolved in the infant within six months of infection and that, ultimately, only 2% SHM is needed to achieve the full breadth of the mature antibody. Mutagenesis and structural analyses reveal that, for this infant bnAb, substitutions in the kappa chain were critical for activity, particularly in CDRL1. Overall, the developmental pathway of this infant antibody includes features distinct from adult antibodies, including several that may be amenable to better vaccine responses.

## Abstract (web summary)

Development of broadly neutralizing antibodies (bnAb) against HIV-1 in infected adults is a multi-step process unachievable by current vaccine approaches. Here the authors reconstruct the ontogeny of an infant bnAb, which develops in fewer steps, and identify its unique features that may shorten the path to HIV vaccines.

## Introduction

Considerable efforts are spent on defining evolutionary pathways of broadly neutralizing antibodies (bnAbs) in HIV-1 infection under the premise that these pathways will help guide effective immunization strategies^1^. Particular emphasis has been placed on bnAb epitopes that are common in different individuals^2^, such as the V3-glycan region of HIV-1 envelope (Env)^3^, and much progress has been made toward characterizing the development of V3-glycan bnAbs in adults^4–7^. While the evolutionary pathway of adult bnAbs have been dissected to inform vaccine approaches^2^, significant challenges remain to be addressed for inducing bnAb responses by vaccination. One such challenge is that most adult bnAbs take years to develop as a result of a complex interplay between viral escape and antibody maturation^2^ that often leads to extensive somatic hypermutation (SHM), ranging from ~6 to 29% (averaging ~18%) for adult-derived V3-glycan bnAbs^4,6,8–13^. Additionally, the inferred germline precursors of a number of bnAbs lack detectable binding to recombinant HIV envelope and thus require the design of germline^−^targeting immunogens for vaccines^4,5,7,8,14,15^. Furthermore, some bnAbs are limited by autoreactivity^6,8,16^.

Recent studies reveal that infants and children develop bnAb responses at least as commonly, if not more frequently, than adults^17,18^ and that they do so rapidly, within 1–2 years post infection^17^. To begin to characterize these early infant cross-clade neutralizing antibody responses, we previously isolated antibodies from an infant with a rapid and broad plasma nAb response. One infant-derived bnAb, BF520.1, demonstrated cross-clade neutralization breadth and targeted the V3-glycan region of HIV Env. In contrast to most adult-derived bnAbs, BF520.1 has limited SHM (VH = 6.6%, VK = 5% nt)^19^. The infant immune system has many unique features compared to adults^20,21^ but the differences between infant and adult antibody development is not known.

Here, we identify the naive antibody precursor of BF520.1, describe the subsequent evolution of this antibody lineage, and explore the structural basis for HIV binding for the first and currently only infant-derived HIV-specific bnAb that has been described. We find that 2% SHM is sufficient for broad neutralization for this antibody lineage and that substitutions in the first complementarity-determining region (CDRL1) of the kappa chain are critical for neutralization breadth. Furthermore, we report that the inferred naive ancestor of this bnAb lineage binds to HIV Env. We conclude that this infant bnAb has features desirable for vaccine design and that further study of infant antibody responses is merited.

## Results

### Ontogeny of the infant-derived bnAb BF520.1

Infant BF520 was infected with clade A HIV, first detectable at 3.8 months of age, and a broadly neutralizing V3-glycan-directed monoclonal antibody (mAb) BF520.1 was isolated ~1–6 years post infection (pi)^19^ at 15 months of age. To infer the ontogeny of this bnAb, we performed next generation sequencing of the infant’s B-cell repertoire on an available post infection sample from ~6 months pi (9 months of age). Full sequencing details are presented in Supplementary Table 1 and Methods. We inferred the naive heavy-chain (VH) and light-chain (VK) ancestors of the BF520.1 mAb, including inference of a previously undocumented variant of VH1-2*02 (G36A) among the per-sample germline results (Supplementary Table 2), and identified all clonally related sequences that descended from these ancestors (i.e., the clonal family of the bnAb) using the partis software package^22–24^. Partis is more accurate than previous methods but has not been previously applied to deep-sequencing bnAb inference.

**Fig. 1:**
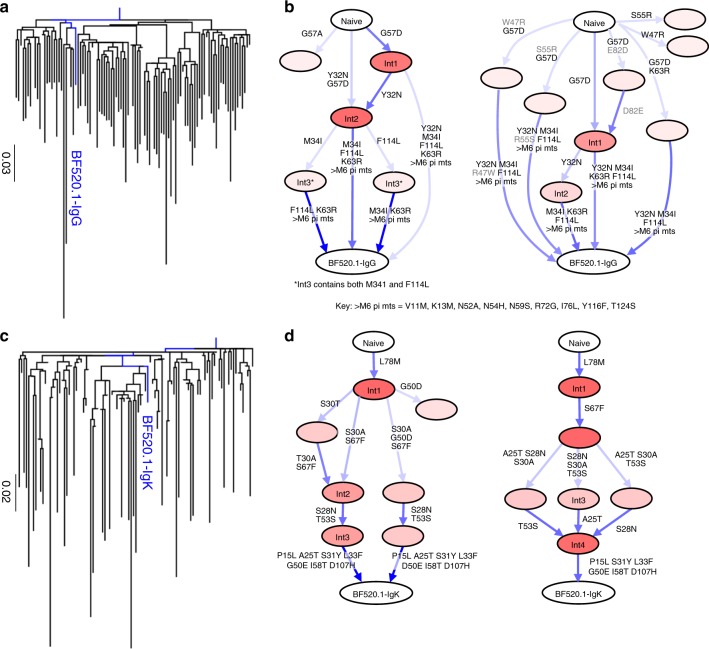
Ontogeny of the infant-derived bnAb BF520.1. **a**, **c** Maximum likelihood phylogenetic relationships of **a** heavy- and **c** light-chain antibody gene variable regions. Trees display the inferred naive ancestor (root), BF520.1 from M12 pi (blue), and representative clonal family member next generation sequencing (NGS) reads from M6 pi (black). Units for branch length estimates are nucleotide substitutions per site. **b**, **d** Most probable routes of BF520.1 development: Bayesian BF520.1 clonal family phylogenies were sampled from an associated posterior distribution and then summarized to display relative confidences for internal node sequences. Resulting graphics display multiple possible lineages of amino acid transitions and their relative confidences for **b** heavy- and **d** light-chain development determined from two NGS technical replicates (left, right). Amino acid substitutions (arrows) connect the inferred naive sequence to the mature BF520.1 sequence via reconstructed ancestral intermediate sequences (nodes). Labeled nodes were chosen as the most probable Bayesian lineage intermediate sequences. The red shading of nodes is proportional to the posterior probability that this ancestral sequence was present in the lineage. For a given node, the blue shading across arrows arising from that node is proportional to the corresponding transition probability. Transient mutations are labeled in gray

To illustrate where BF520.1 falls within the context of its clonal family, maximum likelihood (ML) phylogenetic trees were inferred for VH and VK clonal families (Fig. 1a, c). To reconstruct the most probable developmental routes taken by BF520.1 VH and VK sequences during the first 6 months of infection, we performed Bayesian phylogenetic and ancestral sequence inference analyses for each antibody chain. Importantly, this approach allowed us to rigorously estimate developmental route uncertainty because it accounts for phylogenetic uncertainty by aggregating ancestral inferences from many different phylogenies, which ML does not. We computationally validated that Bayesian lineage reconstruction correctly identified ancestral sequences for simulated antibody lineages with similar characteristics to that of BF520.1 97% of the time for ancestral sequences with posterior probability > 0.8, while dnaml and dnapars were less accurate (87% and 89%, respectively) (Supplementary Fig. 1). Therefore, inspired by Gong et al.’s^25^ approach for reconstructing influenza evolution, we developed a software pipeline that uses Bayesian phylogenetics^26,27^ to reconstruct BF520.1 VH and VK lineages while retaining relative confidences for internal node sequences (Fig. 1b, d). We developed these methods specifically to account for the fact that we had sparse data, in this case from a single sample with limited volume from an infant.

Using two merged replicates of NGS data, we selected the most probable routes of development for the BF520.1 heavy and light chains based on Bayesian lineage inference between infection and 6 months pi. We synthesized chosen lineage intermediates for further study: VH intermediates 1–3 (Int1–3_VH_) and VK intermediates 1–4 (Int1–4_VK_). Selection of these lineages relied on considering the most confident transitions (Fig. 1b, d; darkest blue arrows) from the naive sequence to the mature BF520.1 sequence in both NGS replicates. For the heavy chain, both replicates indicated that the G57D substitution occurred first and thus Int1_VH_ included this change. The Y32N amino acid substitution occurred next in both replicates and was, therefore, incorporated in Int2_VH_. The M34I and F114L substitutions were inferred in one of the two replicates, but the order was unclear, so both were added to Int3_VH_. For the kappa chain, both replicates supported L78M as the first mutation, so this substitution was added to create Int1_VK_. However, there was less agreement between replicates on the chronological order of the substitutions that followed. Both agreed that two substitutions (S30A and S67F) occurred prior to two later substitutions (S28N and T53S), so these steps were incorporated as Int2_VK_ and Int3_VK_. Finally, the A25T substitution was incorporated into Int4_VK_ because one of the two replicates indicated that this was a possible late substitution. Using this approach, we reconstructed highly probable routes of heavy- and light-chain antibody development within the first 6 months of HIV infection. Only naive_VK_ and Int1_VK_ sequences were found at 100% nucleotide identity in the sequencing results.

### VH maturation contributes to heterologous neutralization

**Fig. 2:**
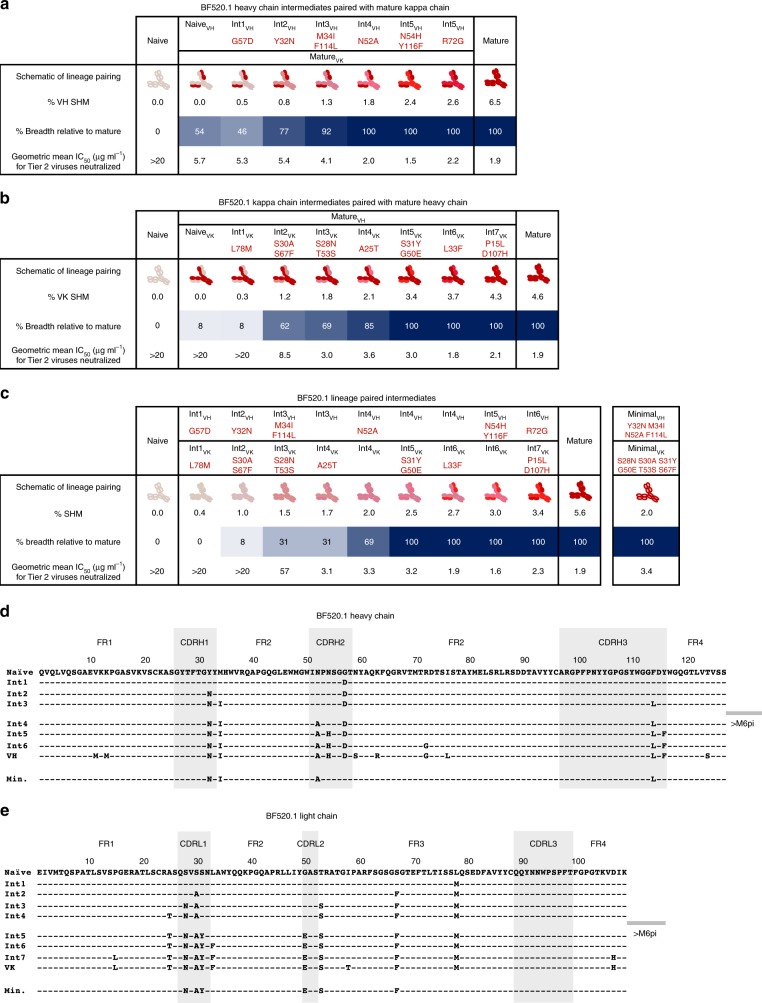
Development of heterologous neutralization by the mature BF520.1 lineage. **a**–**c** Abilities of BF520.1 inferred heavy chain (**a**), light chain (**b**), and paired (**c**) lineage intermediates to neutralize a multi-clade and multi-tier panel of 13 viruses, all of which are neutralized by mature BF520.1. Heavy- and light-chain pairings are indicated along the top row (black text) along with progressive amino acid substitutions (red text). Schematics depict antibody lineage pairings with darker red shades indicating greater maturity. Breadth percentages refer to neutralization of viruses within the 13-virus panel with IC_50_ values of < 20 µg ml^−1^; darker blue shades indicate increased breadth. SHM percentages refer to nucleotide mutation. Geometric mean IC_50_ values (in µg ml^−1^) refer to Tier 2 viruses only. **d**, **e** Amino acid alignment of BF520.1 naive, Bayesian (0–6 months pi) and rationally inferred (6–12 months pi) lineage intermediates, mature, and minimally mutated heavy (**d**) and light (**e**) chain sequences

Antibodies were tested for HIV neutralizing activity against a panel of heterologous viruses that were selected from both the virus panel used to describe infant plasma neutralization breadth^17^ and the standardized “global panel”^28^ based on their ability to be neutralized by the mature BF520.1 antibody with an IC_50_ < 20 μg ml^−1^(ref. ^19^). The inferred naive mAb (naive_VH_naive_VK_) did not demonstrate neutralizing activity against any virus (Fig. 2a and Supplementary Fig. 2a). In contrast to the naive mAb, the naive VH paired with the mature VK (naive_VH_mature_VK_) demonstrated cross-clade tier 2 neutralizing activity (Fig. 2a and Supplementary Fig. 2a). The Int1_VH_mature_VK_ mAb had comparable activity to the naive_VH_mature_VK_ mAb, suggesting that the CDRH2 G57D substitution in this Int1_VH_ did not confer increased neutralizing activity (Fig. 2a, d and Supplementary Fig. 2a). Subsequent VH intermediates demonstrated increasing heterologous, cross-clade neutralizing activity with affinity maturation. Notably, a Y32N substitution in the CDRH1 conferred increased neutralization breadth for Int2_VH_ and further cross-clade breadth and potency was observed in Int3_VH_ with FR2 M34I and CDRH3 F114L substitutions (Fig. 2a, d and Supplementary Fig. 2a).

As the BF520.1 clonal family sequences from M6 pi only informed the first 6 months of BF520.1 antibody development, for mutations that occurred between M6 and M12 pi, we incorporated mutations based on those most likely to confer activity: CDR mutations, CDR-adjacent framework (FR) mutations, non-conservative FR mutations (Int4-6_VH_) (Fig. 2d). With the addition of N52A in the CDRH2 of Int4_VH_, the antibody gained breadth and potency comparable to the mature mAb BF520.1 (Fig. 2a, d).

Previous studies inferred intermediate sequences of antibody lineages from single ML and parsimony (Pars) phylogenies^6,29–31^ and thus did not consider relative confidence as we do with our Bayesian approach. Given this precedent, we compared our results using Bayesian methods against corresponding single ML (Supplementary Fig. 2b, c) and Pars phylogenies (Supplementary Fig. 2d, e) to infer BF520.1 intermediates. Interestingly, the VH intermediates inferred from the ML phylogeny included substitutions that were not found in either the naive or mature mAbs, indicating that they were likely erroneous. The Bayesian lineages also included substitution reversions, but these paths consistently had lower posterior probabilities than paths lacking these artifacts (Fig. 1b, d). When present, these substitutions decreased neutralizing activity, which is apparent when comparing ML Int3_VH_ and ML Int4_VH_ (Supplementary Fig. 2b, c). These findings suggest that the Bayesian antibody lineage determination that incorporated relative confidence over a number of possible lineage pathways prevented the inference of artifactual transient mutations in antibody intermediates. Regardless, our studies of the ML and Pars lineage intermediates led to similar conclusions about the importance of heavy-chain substitutions: CDRH1 Y32N and CDRH2 N52A substitutions resulted in increased neutralization breadth (Supplementary Fig. 2b, c). The contribution of the Y32N CDRH1 substitution was particularly apparent when comparing Pars Int3_VH_, which lacks Y32N and Pars Int4_VH_ (contains Y32N) (Supplementary Fig. 2d, e).

Overall, these data demonstrate increasing heterologous neutralizing activity with affinity maturation and show that the heavy-chain CDRH1 Y32N and CDHR2 N52A substitutions are important for the neutralization breadth of BF520.1.

### VK maturation drives binding and neutralization

**Fig. 3:**
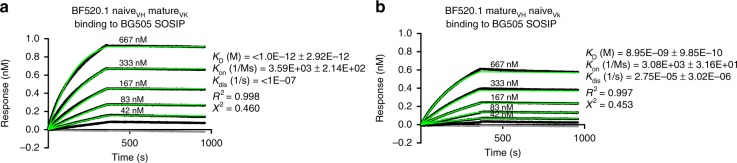
Contribution of kappa chain maturation to HIV binding. **a**, **b** BLI representative reference-subtracted sensorgrams for each interaction between the BG505.SOSIP.664 (ligand) and BF520.1 naive_VH_mature_VK_ (**a**) and mature_VH_naive_VK_ (**b**) (analytes). IgG concentrations ranged from 667 to 42 nM. The gray lines show 0 μM IgG. *K*_D_, *K*_on_, and *K*_dis_ are shown from best fitting (green lines) to a 1:2 bivalent analyte model of binding

Given the surprising finding that the naive VH paired with the mature VK (naive_VH_mature_VK_) demonstrated cross-clade tier 2 HIV neutralizing activity and the naive mAb did not (Fig. 2a), we also examined the evolution of VK in relation to binding of HIV Env and neutralization breadth. The cross-paired mature and naive heavy and light chains (naive_VH_mature_VK_ and mature_VH_naive_VK_) both bound the BG505 SOSIP trimer, which is an Env that is sensitive to BF520.1 neutralization (Fig. 3). The naive_VH_mature_VK_ mAb demonstrated stronger binding to the BG505.SOSIP.664 trimer (*K*_D_ = < 0.001 nM) (Fig. 3a) than the converse mature_VH_naive_VK_ mAb (*K*_D_ = 8.95 nM) (Fig. 3b). In contrast to the heterologous neutralizing activity seen for the naive_VH_mature_VK_ mAb, the mature_VH_naive_VK_ mAb neutralized only tier 1A SF162 (Fig. 2b). These data suggest that maturation in VK is necessary for BF520.1 heterologous neutralization breadth.

Overall, VK lineage intermediates demonstrated increasing breadth with maturation (Fig. 2b and Supplementary Fig. 3a). A dramatic jump in neutralization breadth was demonstrated by Int2_VK_, which contains CDRL1 S30A and FR3 S67F substitutions (2b,e and Supplementary Fig. 3a). As S67F did not increase neutralization breadth (Supplementary Fig. 3b–e), this suggests that the S30A CDRL1 substitution alone enables the observed heterologous neutralization breadth. Further augmentation of potency and breadth was conferred by substitutions observed in Int3_VK_ (CDRL1 S28N and CDRL2 T53S): the T53S substitution did not increase breadth but did confer a slight increase in potency and Int4_VK_ (FR1 A25T) had a very modest increase in activity. Importantly, Int5_VK_ (CDRL1 S31Y and CDRL2 G50E) neutralized all viruses that were neutralized by the mature antibody and Int6_VK_ (FR2 L33F) reached potency comparable to BF520.1 (Fig. 2b, e).

While there are multiple mutations in and around CDRL1, the BF520.1 VK does not contain CDRL3 mutations, based on the inferred naive sequence. These data suggest that kappa chain mutations in and around the CDRL1 are important for BF520.1 heterologous neutralization breadth.

### Cross-clade neutralization achieved with limited SHM

To define the minimal combination of changes in VH and VK that are needed for breadth, intermediates were paired together using percentage SHM to assign probable pairings. Tier 1A neutralizing activity was observed following just two amino acid substitutions in VH (Int2_VH_) and three in VK (Int2_Vk_) (Fig. 2c and Supplementary Fig. 4a) with the additions of the VH Y32N and VK S30A being important for the neutralizing activity. Cross-clade heterologous neutralization was achieved with the addition of the VH M34I and VK S28N in the Int3_VH_3_Vk_ mAb, which had a total of four VH and five VK amino acid substitutions or 1.3% and 1.8% SHM at the nucleotide level in VH and VK, respectively. Int4_VH_ paired with Int4_VK_ neutralized the majority of the viruses (9/13; 69%) that are neutralized by mature BF520.1 with only 1.8% VH and 2.1% VK SHM. Further increased neutralization was conferred by single additional substitutions in both VH and VK: N52A (Int4_VH_) and A25T (Int4_VK_). The breadth of BF520.1 was reached with 1.8% VH and 3.4% VK SHM (2.5% overall SHM) for Int4_VH_5_VK_. Finally, the Int5_VH_6_VK_ mAb reached the breadth and potency of mature BF520.1 with only 2.4% VH and 3.7% VK SHM (3.0% overall SHM) (Fig. 2c and Supplementary Fig. 4a). Similar observations were made using the ML (Supplementary Fig. 4b) and Pars (Supplementary Fig. 4c) lineages.

We used results from the analyses above, suggesting that CDRH2 G57D, FRL1 A25T, and FRL3 L78M substitutions were not critical to BF520.1 function, to construct an antibody with the minimal set of mutations implicated in broad activity. This minimal BF520.1 antibody, which has and overall SHM of 2% (1.3% nt in Minimal_VH_ and 2.7% nt in Minimal_VK_), exhibited comparable breadth and potency to the mature bnAb (Fig. 2c and Supplementary Fig. 4a), thus defining the minimal mutations for the activity of this antibody.

### Cryo-EM structure of the HIV Env trimer with BF520.1 Fab

**Fig. 4:**
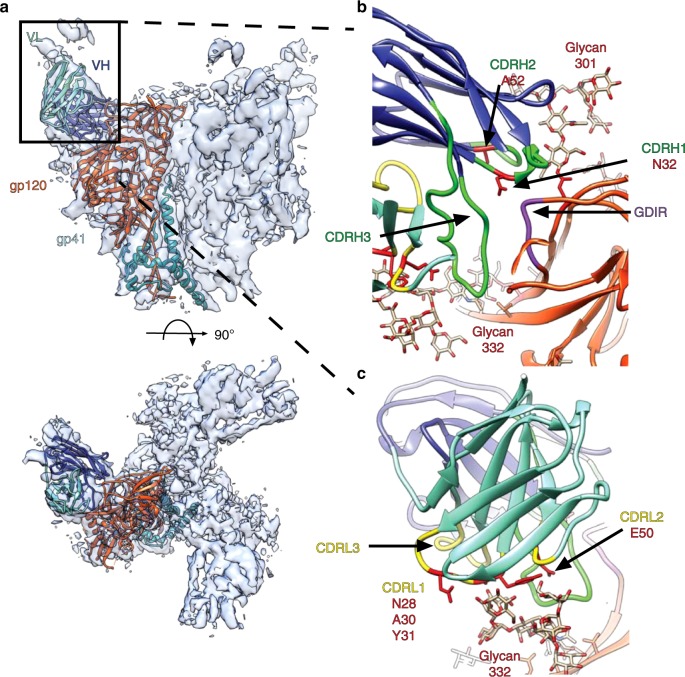
Cryo-EM reconstruction and model of the BG505.SOSIP.664 trimer in complex with BF520.1 Fab. **a** Side (above) and top (below) views of the cryo-EM reconstruction and structural model. A single monomer consisting of gp120 (orange) and gp41 (dark cyan), and the BF520.1-Fv variable heavy chain (dark blue) and variable light chain (aquamarine) are highlighted. Glycans removed for clarity. The BG505.SOSIP trimer structure 5ACO.pdb docked into the new EM density map with a correlation score of 0.8602^34^. A global search yielded a preferred placement of the BF520.1 Fv model into the Fab density with a correlation score of 0.8513 (Supplementary Fig. 5g). **b** Expanded view of the VH domain and gp120. Shown are the conserved gp120 GDIR sequence (purple), glycans N332 and N301, and CDRH loops (green). Mutations that confer potent neutralization (red) are indicated. **c** Expanded view of the VL domain and the N332 glycan. CDRL loops (yellow) and mutations that conferred potent neutralization (red) are highlighted

Single-particle cryo-electron microscopy was used to gain insight into the interaction of BF520.1 Fab and BG505.SOSIP.664 trimer, which is a clade A transmitted envelope variant from a Kenyan infant who was in the same cohort as BF520^32,33^. The resulting 4.8 Å map (Fig. 4 and Supplementary Fig. 5) revealed features consistent with the resolution estimate including helices, beta-sheet structure, as well as bulky densities for glycans that protrude from glycosylation sites, including N332 and N301, which are critical features for BF520.1 activity^34–37^ (Supplementary Fig. 5e).

The positioning of the CDRH loops in our structural model indicates that they make multiple contacts with both protein and N-linked glycans (Fig. 4b). The CDRH1 loop is in close proximity to the base of the gp120 V3-loop, with N32 oriented towards the conserved GDIR sequence, consistent with increased neutralization observed when asparagine is introduced at position 32 on the CDRH1 loop (Y32N substitution). Adjacent residues of CDRH1, as well as the CDRH2 loop are located proximal to the N301 glycan. It is unclear if residue 52 (N52A substitution) in CDRH2 mediates direct contact between either the GDIR sequence or N301 glycan; however, it is plausible that substitution for alanine could influence loop positioning such that residues H54 and S55 would interact with the N301 glycan. The relative orientation of the CDRH3 loop suggests it may interact with the V3 GDIR sequence, as well as the N332 glycan, though given variability in CDRH3 positioning between homology models, the contacts are less certain for this loop.

The structure of the Env trimer in complex with BF520.1 supports a role for the BF520.1 VK in neutralization through extensive contacts between the CDRL1 loop and the N332 glycan. Based upon the BF520.1 homology model, N28, A30, and Y31, which were implicated in nAb breadth, are positioned directly adjacent to the N332 glycan (Fig. 4c), although we cannot unambiguously identify the position of specific residues. L33F and G50E mutations contributed to increased potency, but from the structure it is unclear whether these residues could directly contact the N332 glycan or protein surface^38,39^; they may instead alter local paratope structure in a more indirect fashion. The position of CDRL3 loop suggests it likely does not interact with the BG505 trimer or key glycans, which may explain why it does not contain mutations that contribute to BF520.1 breadth.

### BF520.1 naive and lineage members bind HIV Env trimer

**Fig. 5:**
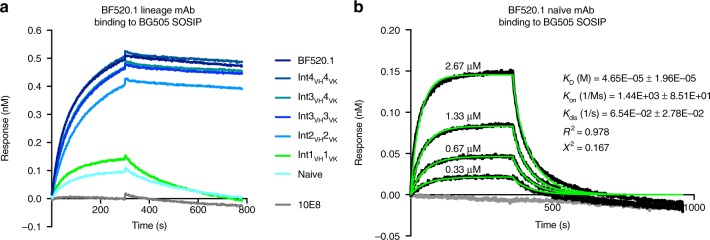
BF520.1 naive and lineage binding to HIV Env trimer. **a** BF520.1 paired lineage intermediates (ligand) binding to the BG505.SOSIP.664 (analyte) measured by BLI. 10E8 is a negative control as the SOSIP trimer does not contain the targeted MPER epitope. Data are representative of two independent experiments. **b** BLI-binding analysis of varying concentrations of BF520.1 naive antibody (analyte) binding to BG505.SOSIP.664 (ligand). The gray line shows 0 μM IgG. *K*_D_, *K*_on_, and *K*_dis_ from best fitting (green lines) to a 1:2 bivalent analyte model of ligand:analyte binding are shown

Although the inferred naive ancestor of BF520.1 did not demonstrate neutralizing activity, it weakly bound to BG505.SOSIP.664 trimer (Fig. 5a, b) and this binding was dependent on increasing mAb concentration (Fig. 5b). Qualitatively, we observed increased binding with affinity maturation (Fig. 5a) with the most significant increase in binding demonstrated by Int2_VH_ Int2_VK_ and more subtle increases by later intermediates. Mutations acquired by Int4_VH_4_VK_ enabled similar binding to Env as the mature BF520.1. Despite clear binding to the BG505 SOSIP trimer by Int2_VH_2_VK_, Int3_VH_3_VK_, and Int3_VH_4_VK_, these intermediates were not able to neutralize the BG505 pseudovirus (Figs. 2c and 5a). These data indicate that the BF520.1 naive mAb had the potential to recognize HIV Env.

Our approach to inferring the naive ancestor using replicate sequence data differed from previous studies, which only used single replicates of NGS data^6,29,30^. To compare the two methods, we also inferred the BF520.1 naive ancestor using just the first replicate of antibody sequence data. This approach resulted in uncertainty in the naive nucleotide sequences at three positions (Supplementary Fig. 6a, b) while there was not uncertainty using replicate datasets. When tested, none of the alternative inferred naive mAbs demonstrated neutralization (Supplementary Fig. 6c), nor did they bind trimer (Supplementary Fig. 6d), which contrasts the ability of the more probable replicate-inferred naive mAb. We also found differences in the autoreactivity of the inferred naive mAbs: single dataset-inferred mAbs demonstrated weak autoreactivity (Supplementary Fig. 6e), whereas this was not observed for the more probable replicate-inferred naive mAb. These data demonstrate that uncertainty in the inference of bnAb precursors can result in significant differences in HIV Env binding and autoreactive properties.

### Increasing autologous binding and neutralizing by lineage

BF520.1 binds to but does not neutralize the transmitted autologous virus and neutralizes variants isolated from 6 months of age (M6), which is ~2.2 months after infection was detected^19^. The naive and earliest intermediates (mAbs Int1_VH_1_VK_, Int2_VH_2_VK_, Int3_VH_3_VK_, Int3_VH_4_VK_, and Int4_VH_4_VK_) did not demonstrate binding to or neutralization of the transmitted autologous virus (Supplementary Fig. 7). Likewise, these early paired intermediates failed to bind and neutralize the M6 variants, with the exception of Int4_VH_4_VK_, which bound weakly to BF520.M6.D1 (Supplementary Fig. 7) and weakly neutralized BF520.M6.I1 with a lower potency than the mature bnAb (5.8 vs. 1.1 μg ml^−1^, respectively). In contrast, later intermediates (mAbs Int4_VH_5_VK_, Int4_VH_6_VK,_ Int5_VH_6_VK_ and Int6_VH_7_VK_) demonstrated increases in binding and neutralization approaching that of mature BF520.1: they bind autologous variants from transmission and 2.2 months pi, but only neutralize the 2.2 month pi variants. Of note, Int4_VH_5_VK_ and Int4_VH_6_VK_ mAbs demonstrated stepwise increases in neutralization of the autologous viruses from ~2.2 months pi, further highlighting the importance of the kappa chain contribution to autologous neutralizing activity. Substitutions in the CDRL1 and CDRL1-proximal regions were particularly impactful for autologous neutralization: CDRL1 S31Y, CDRL2 G50E and FR2 L33F (Supplementary Fig. 7).

## Discussion

The complex developmental pathways of bnAbs in HIV-infected adults have been carefully examined to guide immunogen design targeting key precursors in antibody evolutionary pathways^2^. In many cases, this approach is complicated by the observation that the inferred naive progenitor of the bnAb does not bind HIV Env, presenting challenges to stimulating the lineage^15^. Taking advantage of the rapid development of HIV bnAb responses in infants, here we discovered an evolutionary pathway that starts with a naive antibody that is capable of binding HIV Env trimer and requires as little as 2% SHM to achieve the full cross-clade breadth of this lineage. We show that both the heavy and light chains, mostly outside of the CDRH3, are functionally important for development of HIV specificity and breadth for this bnAb. Thus, this antibody developmental pathway allows for two key aspects of vaccine design that were stumbling blocks for many adult bnAbs pathways: the HIV antigen can engage the naive B cell and a level of SHM that is easily within reach by vaccination^40^.

V3-glycan targeting bnAbs are of high interest because they are common and not restricted by certain germline genes^4,6,8,10,12,19^. BF520.1 is of particular interest because it developed more rapidly than adult V3-bnAbs. Our study of this infant bnAb’s evolution suggests it achieved broad and potent capability with just 3% SHM and that, upon excluding irrelevant mutations from the lineage, only 2% SHM was required for this functionality. Other known adult V3-glycan bnAbs required rare mutation events to occur^4,7^ and/or higher levels of SHM^6^ compared to BF520.1^41^. The highly impactful N52A substitution in the BF520.1 heavy chain required one nucleotide mutation in an AID hotspot site and one mutation in a neutral site, but the improbability of these mutations was not a barrier to rapid bnAb development in this infant. This indicates that vaccines may be able to stimulate similar mutations in reasonable timeframes.

Interestingly, mutations important for increasing BF520.1 functional activity were primarily found in the heavy-chain CDRH2 and light-chain CDRL1, with little-to-no contribution from the CDRH3 and CDRL3. This contrasts many HIV bnAbs where major determinants of breadth reside in the CDR3 regions, particularly in the heavy chain^42^. The Cryo-EM structural model indicated that CDRH2 and CDRL1 residues appear to contribute to the BF520.1 paratope by mediating contacts with the conserved V3-glycan site, namely the archetypal features of V3-targeting bnAbs: GDIR sequence and glycans at position 301 and 332^43^. The angle of approach by BF520.1 towards the V3 epitope was previously determined to be similar to bnAb PGT128, although BF520.1’s positioning was notably different and slightly rotated relative to PGT128^19^. The crystal structure of PGT128 in complex with gp120 identified the GDIR motif and glycans at positions N332 and N301 as the primary contacts defining the PGT128 epitope^44^. The CDRH3 loop, which is highly mutated in PGT128, penetrates the glycan shield in order to contact both the GDIR motif and N332 glycan. Our structural model similarly indicates potential CDRH3 contacts against the GDIR motif and N332 glycan; however, it is unclear if the lack of mutations during maturation suggest a minor role or if the naive CDRH3 sequence is optimally suited for binding and broad neutralization. For PGT128, less certainty exists about the contribution of the light chain, which is recessed relative to the heavy chain and has been suggested to either allow contacts with high mannose glycans at N137/N156, or to avoid making contacts altogether^34,44^. In contrast, the slight rotation of BF520.1 relative to PGT128 places the CDRL1 loop in closer proximity to the N332 glycan. Each of the three substitutions in CDRL1 (N28, A30, and Y31) are situated to make contacts with N332, which supports increasing breadth and potency along the VK maturation pathway.

Structural studies have shown there are significant CDRL contacts present in adult-derived V3 targeted antibody lineages, including to the N332 glycan and GDIR motif^10,37,45,46^, but, with few exceptions^7^, functional studies have focused on the contribution of VH to neutralizing activity^4,6^. However, recent studies have shown that structurally defined antibody-Env contacts do not fully capture the functional interactions that drive neutralization and escape, reinforcing the importance of functional studies to define the interactions that are critical for neutralization activity^47^. Here, we combined functional and structural approaches and showed that maturation in the BF520.1 VK was functionally important for HIV Env binding and neutralization, demonstrated by the increased heterologous breadth observed when the mature VK was paired with the naive VH and the five amino acid substitutions in and around CDRL1 that contribute to neutralization breadth; findings that were support by the structural analyses. Thus, our studies highlight the importance of considering both the heavy and light chain in the development of HIV-specific bnAbs and the fact that the light chain can potentially be harnessed to develop vaccine approaches that elicit such bnAbs with relatively little SHM.

While we see neutralization of heterologous Env variants by the early BF520.1 lineage intermediates, autologous neutralization is observed only with later stage intermediates (Int4_VH_4_VK_ and beyond) against BF520 autologous Env variants isolated at ~2 months pi. While CDRH2 and CDRL1 mutations are important for heterologous breadth, autologous neutralization was conferred by additional kappa chain mutations in and around the CDRL1. The observation that the earlier intermediates are HIV-specific but do not neutralize the autologous virus suggests that the virus at ~2 months pi may have escaped neutralization by the early lineage. We found that the lineage mAbs were able to bind Env of autologous viruses. Interestingly, Int4_VH_5_VK_ and subsequent mAbs, all of which demonstrate cross-clade breadth and potency, could bind to the transmitted viral Env, BF520.W14.E3. However, Int4_VH_4_VK_, which contains a less-mutated kappa chain, bound the virus from ~2 months pi, but not the transmitted founder, suggesting that this evolved virus contains mutations that allow interaction with earlier intermediates in the antibody maturation pathway. The BF520.1 naive mAb and early lineage intermediates do, however, bind the heterologous clade A BG505.SOSIP.664 trimer, which contrasts with many adult bnAb precursors^15^, including some V3-glycan bnAbs^4,7^. The affinity of the BF520.1 naive mAb for the Env trimer is within the range of affinities observed for VRC01-class naive precursors binding to a monovalent germline-targeting antigen^48^. Based on the binding results with the naive BF520.1 precursor, we hypothesize that the BG505.SOSIP.664 could initiate a BF520.1-like response and this could potentially be boosted with BF520 Env trimer from ~2 months pi, which binds to early intermediates in this lineage to drive affinity maturation.

For vaccine immunogen design, it is critical that inferred bnAb precursor heavy- and light-chain sequences are as accurate as possible. Importantly, accurate naive inference enables more accurate phylogenetic inference of intermediate sequences. By using replicate NGS data, we resolved uncertainties in the inferred naive sequences that impacted the overall characterization of our antibody lineage. We are confident in our BF520.1 naive inference for three reasons: (1) we used the entire clonal family to infer the naive sequence along with more accurate clonal family clustering, which together increase confidence especially within the CDR3^22,23^, (2) we used per-sample germline inference^24^, which mitigates gene assignment errors and expands our ability to accurately infer antibody ontogenies in non-European subjects whose antibody gene usage patterns are less well documented, and (3) BF520.1 had very-low SHM compared to other bnAbs, which alleviated the difficulty of naive inference due to a reduced expected number of incorrect bases. Notably, our most probable replicate-inferred naive mAb measurably bound HIV and did not demonstrate autoreactivity. Conversely, the single dataset-inferred naive mAbs that contained uncertainties displayed autoreactivity, which is relevant because many inferred naive sequences for adult bnAbs^16^ and some V3-glycan bnAbs^6,8^ show autoreactivity. These discrepancies highlight the potential imprecision of naive antibody inference, especially when analyzing sequencing data of insufficient quality or depth, and suggest that our approaches designed specifically for antibody lineage analysis mitigate some of these problems.

We developed and validated inference methods tailored to sparse data, which is a natural consequence of sample age and small sample volume. Indeed, infants present unique challenges for such detailed studies of their antibody responses because prevention of mother-to-child transmission and early infant treatment have been so successful. Thus, these types of studies have limitations because they require decades-old stored samples and few are available. Moreover, the volume of sample available from infants is small. These limitations, along with the large size and highly variable dynamics of the B-cell repertoire^49,50^, may explain why we did not detect many of the inferred intermediates within the deep-sequencing dataset. The lack of detection of the inferred intermediates should be considered in the context of our motivation for interrogating the inferred antibody maturation in the infant, which was not to define with certainty how the BF520.1 antibody evolved in this one individual, but rather to use the probable pathway in the infant to define how a minimally mutated broad and potent antibody lineage could arise more generally. Likewise, our analyses with paired heavy- and light-chain intermediates was not designed to perfectly reflect paired intermediates found in infant BF520, but rather to identify key mutations that drive breadth using pairs that are more relevant than pairing intermediates with mature or naive chain partners. This approach was important in identifying single amino acids such as VH N52A and VK A25T as critical drivers of breadth and potency.

The finding that infants develop broad and potent HIV bnAb responses has raised the question of whether HIV vaccine efforts should focus on infants^20^, which is appealing because infants and children have regular contact with the healthcare system. Given that the BF520.1 naive ancestor binds HIV Env, that very little SHM is required for this V3-directed antibody lineage to demonstrate heterologous cross-clade breadth, and that these antibodies lack unusual or rare features, the BF520.1 lineage should be considered an attractive template for vaccine design. These findings also raise the interesting possibility that there are unique features of the infant response, such as the naive repertoire or other immunological aspects of infant immunity, that could uniquely position the infant immune system to develop bnAb and other antibody responses rapidly.

## Methods

### Human subjects

Peripheral blood mononuclear cell (PBMC) samples were obtained from infant BF520 enrolled in the Nairobi Breastfeeding Clinical Trial^32^, which was conducted prior to the use of antiretrovirals for prevention of mother-to-child transmission. Approval to conduct the Nairobi Breastfeeding Clinical Trial was provided by the ethical review committee of the Kenyatta National Hospital Institutional Review Board, the Fred Hutchinson Cancer Research Center Institutional Review Board, and the University of Washington Institutional Review Board.

### Sample preparation and RNA isolation

PBMCs stored in liquid nitrogen for ~20 years were thawed at 37 °C, diluted tenfold in pre-warmed RPMI and centrifuged for 10 min at 300 × *g*. Cells were washed once in phosphate-buffered saline, counted with trypan blue, centrifuged again, and total RNA was extracted from PBMCs using the AllPrep DNA/RNA Mini Kit (Qiagen), according to the manufacturer’s recommended protocol. RNA was stored at −80 °C until library preparation.

We performed library preparation, sequence analysis, and antibody lineage reconstruction in technical duplicate, using the same RNA isolated from each time-point, week 1 (W1) and month 9 (M9). We merged the sequences from our NGS replicates to achieve more depth after discovering low overlap between the datasets, indicating that we had not saturated the repertoire even though the sample was exhausted.

### Antibody gene deep sequencing

Antibody sequencing was performed as described^51^. Briefly, RACE-ready cDNA synthesis was performed using the SMARTer RACE 5′/3′ Kit (Takara Bio USA) using primers with specificity to IgM, IgG, IgK and IgL. We did not identify any BF520.1-clonal IgM sequences; IgM and IgL were used for germline Ig gene inference. The cDNA was diluted in Tricine-EDTA according to the manufacturer’s recommended protocol. First-round Ig-encoding sequence amplification (20 cycles) was performed using Q5 High-Fidelity Master Mix (New England BioLabs) and nested gene-specific primers (Table 1). Amplicons were directly used as templates for MiSeq adaption by second-round PCR amplification (20 cycles). Amplicons were then purified and analyzed by gel electrophoresis and indexed using Nextera XT P5 and P7 index sequences for Illumina sequencing according to the manufacturer’s instructions (ten cycles). Gel-purified, indexed libraries were quantitated using the KAPA library quantification kit (Kapa Biosystems) performed on an Applied Biosystems 7500 Fast real-time PCR machine.

**Table 1:** Illumina Miseq library preparation primers

Conc. (nM)	Primer ID	Sequence
	RT-PCR	
500	3′IgM (OUTER)	CCACTTCGTTTGTATCCAACG
500	3′IgG (OUTER)	GCCGGGAAGGTGTGCACGCCGCTGGTC
500	3′IgK (OUTER)	GTCCTGCTCTGTGACACTCTC
500	3′IgL (OUTER)	TGTTGCTCTGTTTGGAGGG
	PCR 1	
800	3′IgM (INNER)	GCATTCTCACAGGAGACGAGG
800	3′IgG (INNER)	CCGGTTCAGGGAAGTAGTCCTTGAC
800	3′IgK (INNER)	ATTCAGCAGGCACACAACAGAGGC
800	3′IgL (INNER)	AGACACACTAGTGTGGCCTTG
800	vv535: Step out primer	GACAAGCAGTGGTATCAACGCAG
	PCR 2	
400	*MiSeq* HsRh IgM CH1 R	*GTCTCGTGGGCTCGGAGATGTGTATAAGAGACAG*GGGTTGGGGCGGATGCACT
400	*MiSeq* HsRh IgG CH1 R	*GTCTCGTGGGCTCGGAGATGTGTATAAGAGACAG*GGGGGAAGACCGATGGGCCCTT
400	*MiSeq* HsRh IgK CL1 R	*GTCTCGTGGGCTCGGAGATGTGTATAAGAGACAG*GAAGACAGATGGTGCAGCC
400	*MiSeq* HsRh IgL CL1 R	*GTCTCGTGGGCTCGGAGATGTGTATAAGAGACAG*GGAACAGAGTGACCGTGGG
400	vv539: *MiSeq*-adapted step out primer	*TCGTCGGCAGCGTCAGATGTGTATAAGAGACAG*CACTCTATCCGACAAGCAGTGGTATC

Italicized bases = MiSeq adapter

Libraries were denatured and loaded onto Illumina 600-cycle V3 cartridges, according to the manufacturer’s suggested workflow.

### Sequence analysis and clonal family clustering

Sequences were preprocessed as previously described^51^. Briefly, amplicons were reconstructed from forward and reverse MiSeq reads using FLASH^52^ and the amplification primers were trimmed using cutadapt^53^. Sequences that contained low-confidence base calls (N’s) were removed (FASTX-toolkit). Filtered sequences from both time-points (W1 and M9) were combined and annotated with partis (https://github.com/psathyrella/partis) using the default options (including per-sample germline inference, Ralph in review at PLOS Comp Biol http://arxiv.org/abs/1711.05843). Sequences with internal stop codons, or with CDR3 regions out of frame were removed during this step. We did not exclude singlets in an attempt to retain even very rare sequences. Sequences were then clustered into clonal families using the seed clustering method^22,23^ and the previously identified BF520.1-IgG and BF520.1-IgK sequences^19^ as cluster “seeds.” This approach also included the inference of the unmutated common ancestor sequence for each clonal family. Sequencing results and clonal clustering summaries are detailed in Supplementary Table 1. We found 1126 clonal gamma sequences and 5174 clonal kappa sequences. Most BF520.1-clonal sequences were singlets, but we could not distinguish biologically accurate singlets from those that contained PCR- or sequencing errors. Thus, we erred on the side of keeping all sequences and, instead, mitigated the impact of individual sequencing errors by using Bayesian lineage inference on the BF520.1 clonal families. Also of note, due to light-chain rearrangement mechanics, it is common for clustering analyses to include an overabundance of clonal sequences that may not have originated from the same rearrangement event, potentially explaining the larger number of clonal kappa sequences found than gamma sequences. This caveat is inherent to all clonal clustering strategies yet developed and ultimately reduces accuracy in inferring true maturational pathways.

### Antibody lineage reconstruction

Initial phylogenetic trees of BF520.1 heavy- and light-chain clonal families were inferred using FastTree^54^ and pared down to the 100 sequences per chain that were the most relevant to inferring the lineage history of BF520.1 based on proximity to nodes within the “seed lineage” extending from the inferred naive ancestor (root) to the mature BF520.1 heavy or light sequence (prune.py, https://git.io/fh94Y). The prune.py program cycles along the path from seed to naive ancestor, adding closest sequences to that path until it achieves the target number of sequences. The 100 clonal sequences were then analyzed by BEAST^26^. BEAST output was summarized for internal node sequences using the approach presented by Gong et al.^25^. In the resulting summary graphic, each red oval represents a unique inferred sequence, with color intensity proportional to the relative confidence that the true-lineage included that intermediate. Arrows correspond to amino acid substitutions, with color intensity proportional to the relative confidence that this specific substitution occurred. The most probable BF520.1-IgG lineage paths were consistent between both technical replicates. For BF520.1-IgK, replicates agreed on probable lineage paths but each replicate offered higher resolution at a different part of the lineage (early mutations vs. later mutations). These data were combined to estimate the M0-M9 BF520.1-IgK lineage.

In addition to Bayesian lineage analysis, maximum likelihood (dnaml) and maximum parsimony (dnapars) phylogenetic trees were inferred from the list of 100 clonal sequences, including ancestral sequence reconstruction using PHYLIP tools (http://evolution.genetics.washington.edu/phylip/getme-new1.html) (Felsenstein, J. 2017. PHYLIP (Phylogeny Inference Package) version 3.696. *Distributed by the author. Department of Genome Sciences, University of Washington, Seattle*). Trees were rooted on the inferred naive sequence and we identified intermediate sequences that were found on lineage paths between naive to seed sequence. For dnaml and dnapars analyses, filtered IgK sequences were down sampled to 100,000 randomly selected sequences prior to partis seed partitioning and FastTree trees were pared down using an earlier version of prune.py (https://git.io/fh943).

### Validation of Bayesian lineage reconstruction

To computationally validate the performance of Bayesian ancestral sequence inference in antibody lineage reconstruction, we simulated 10 independent clonal families that had similar characteristics to the BF520.1 clonal family and performed Bayesian inference as described in this study on these simulated clonal families. Even though we exclusively used BEAST to perform Bayesian ancestral sequence inference on the BF520.1 clonal family, we additionally performed validation experiments using another popular Bayesian inference software package RevBayes^27^. RevBayes outputs trees that have unconstrained branch lengths, unlike BEAST, which outputs trees with fixed sampling time(s); in particular, RevBayes uses a Uniform prior on tree topologies and Exponential priors on branch lengths, while BEAST uses a birth-death process model as a topology prior and a strict-clock prior on branch lengths. For all MCMC analyses, we used 10% burnin and ran the chains for 10,000,000 iterations. Clonal family simulation was performed using the procedure described in Davidsen and Matsen^55^. In short, this procedure approximates germinal center dynamics via nucleotide-context-sensitive mutation and amino-acid selection components and intermediate sampling times. We adjusted the simulation parameters such that the simulation produced clonal families similar to the BF520.1 family in terms of the distribution of root-to-tip distances. To do so, we computed the distribution of the median root-to-tip branch length distances from 10,000 independent RevBayes tree samples for all sequences in the BF520.1 family and matched this distribution with the corresponding distributions from our simulated clonal families (Supplementary Fig. 1b). The simulated “seed” sequence (meant to mimic a sequence of particular interest) was chosen to be the sequence farthest from the root naive sequence.

To assess the accuracy of BEAST and RevBayes in antibody lineage inference, reconstructed ancestral sequences (on the lineage from the naive sequence to the simulated “seed” sequence) were compared to sequences present on the known “true” lineage ancestral sequences in simulated phylogenies. We also ran dnaml and dnapars on these simulated families to compare accuracy between these four methods (Supplementary Fig. 1a). For the Bayesian methods, the posterior probability of a sequence (which we call P(ASR) here for short) is calculated as the number of occurrences of that sequence divided by the number of posterior samples. Any true-lineage sequence that didn’t appear in the posterior was assigned P(ASR) = 0. For ML and parsimony, if a sequence was found in the reconstruction, P(ASR) was taken to be 1, and any true-lineage sequence not in the reconstruction got P(ASR) = 0. We repeated simulation 10 times with the same parameters, chosen to mimic the BF520.1 clonal family as described above. The results were then collated across simulations to form a single accuracy table, (Supplementary Fig. 1a). Thus, *N* is the total number of unique reconstructed and “true” lineage sequences across the simulations for a given method. Although BEAST showed slightly better overall performance and we decided to use it for this analysis, RevBayes also resulted in highly accurate reconstruction.

Our analysis pipeline for the Bayesian phylogenetics component of this work is available at https://github.com/matsengrp/ecgtheow for the purpose of reproducibility. We are currently working on a software tool to enable a more sophisticated version of the analysis presented here.

### VH and VK lineage intermediate pairing

M0-M6 pi intermediates (Bayesian lineage Ints1–3_VH_ and Ints1–4_VK_, Figs. 2b and 3d) were paired together using percentage SHM to assign probable pairings. The remaining post-M6 pi intermediates were paired based on increasing SHM and their incorporation of additional CDR mutations, CDR-adjacent FR mutations and non-conservative FR mutations. Pars and ML lineage intermediates were also paired using percentage SHM to assign probable pairings. As the M6-M12 pi rationally inferred substitutions would be the same for ML and Pars lineages, these additional changes were added to the Pars lineage only.

### mAb preparation

Antibody heavy- and light-chain variable regions were synthesized as “gBlocks” by Integrated DNA Technologies (www.idtdna.com) and subsequently cloned into IgG and IgK expression vectors as previously described^56^. Equal ratios of heavy- and light-chain plasmids were co-transfected into 293F cells using FreeStyle MAX (Invitrogen) according to the manufacturer’s instructions. Protein G columns were used to purify IgG as previously described^57^.

### Pseudovirus production and neutralization assays

Pseudovirus production and neutralization assays were performed as previously described^58^ with an alternative cell lysis and B-galactosidase detection system (Gal-Screen; ThermoFisher). Specifically, 85 μl of the 150 μl total volume was removed from each well (50 μl remaining) and 50 μl of Gal-Screen substrate (diluted 1:25 in “Buffer A”) was added to each well. Luminescence was measured after 40 min incubation at room temperature. The mAbs were diluted twofold from 20 to 0.6 μg ml^−1^. IC_50_ values represent the concentration (μg ml^−1^) at which 50% of the virus was neutralized and are the average of two or three independent experiments performed in duplicate.

### Biolayer interferometry assays

Binding of mAbs to HIV Env SOSIP trimers was measured using biolayer interferometry on an Octet RED instrument (ForteBio). For the qualitative comparison of lineage intermediate binding, IgG antibodies diluted to 10 μg ml^−1^ in PBS plus 1% BSA, 0.01% TWEEN-20, and 0.02% sodium azide were immobilized onto anti-human IgG Fc capture (AHC) biosensors and BG505.SOSIP.664 (diluted to 1 μM in the same buffer) was flowed as analyte in solution. For mAb kinetic determination, BG505.SOSIP.664-AviB (25 μg ml^−1^) was immobilized onto Streptavidin (SA) biosensors. Varying concentrations of IgG were flowed as analyte in solution. A series of six, twofold dilutions of naive_VH_mature_VK_ and mature_VH_naive_VK_ mAbs (667 to 21 nM), as well as 0 nM IgG were tested. The BF520.1 naive mAb concentrations ranged from 2.67 to 0.33 μM (series of four, twofold dilutions) and 0 μM. Association was monitored for 6 min and dissociation for 10 min. Binding-affinity constants (*K*_D_; on-rate, *K*_on_; off-rate, *K*_dis_) were determined using ForteBio’s Data Analysis 7.0. Average measurements from reference wells were subtracted and data were processed by Savitzky-Golay filtering prior to fitting using a 1:2 (bivalent analyte) model of binding.

### Cell-surface-binding assays

Binding to cell-surface Env was measured using a flow cytometry-based assay^59^. In all, 293T cells (5 × 10^5^ cells) were transfected with 1.33 µg HIV-1 env DNA and 2.66 µg Q23Δenv, an envelope-deficient proviral plasmid, using Fugene6 (Promega), harvested 72 h post transfection, and incubated with 20 mg ml^−1^ mAb. Cells were then incubated with a 1:100 dilution of goat-anti-human IgG-PE (Jackson ImmunoResearch), subsequently fixed with 1% paraformaldehyde, and processed by flow cytometry using a BD FACS-Canto II. Data was analyzed using FlowJo software. Percent binding was calculated as the percentage of PE-positive cells with background (mAb binding to mock transfected cells) subtracted. Analyses were performed in GraphPad Prism 7.0d.

### Sample preparation for cryo-electron microscopy

Purified BG505.SOSIP.664 trimers^60^ were mixed with fourfold molar excess BF520.1 Fab and diluted to 0.4 mg ml^−1^ in PBS or PBS supplemented with 70 µM n-Dodecyl-$\beta$-d-Maltoside

(DDM). The mixture incubated for 1 h at room temperature prior to vitrification. A 3.0 µl aliquot was applied at 4 °C and 100% humidity to glow-discharged C-Flat 1.2/1.3 4C holey carbon-coated grids (Electron Microscopy Sciences), blotted, and immediately plunge frozen in liquid ethane by using a Vitrobot Mark IV specimen preparation unit (FEI Co.).

### Cryo-EM data collection, refinement, and validation statistics

Vitrified grids were imaged using an FEI Titan Krios operating at 300 keV and equipped with a Gatan K2 summit direct detector device. Micrographs were collected at 130,000 × , corresponding to a pixel size of 0.55 Å pixel^−1^ in super resolution mode. Each image received a dose rate of ~8 e^−^ pixel^−1^ s^−1^ with 200 ms exposure per frame, and an estimated defocus ranging from 1.0 to 3.5 µm. Data were collected in three separate sessions, resulting in a total of 3454 images using EPU (908 micrographs) (FEI) and Leginon (2546 micrographs)^61^ automated data collection softwares.

### Cryo-EM data processing

Frame alignment and CTF estimation were carried out independently for data collected using EPU and data collected using Leginon softwares. Frame alignment and dose-weighting were completed using MotionCor2^62^, and CTF estimation was performed using CTFFIND4^63^. Relion 2.1^64^ was utilized for further processing and three-dimensional (3D) refinement. Approximately 1000 particles were manually selected and subjected to 2D classification to build templates for automated particle picking. A total of 559,022 particles was selected and binned to 8.8 Å pixel^−1^ for expeditious processing. The binned particle stack was subjected to 2D classification where 116,972 were selected for 3D refinement. This particle stack was re-extracted as a 4 × binned stack (pixel size 2.2 Å pixel^−1^). Approximately 32,000 particles were chosen for 3D classification and a subset of ~9000 particles was selected to build a low-resolution 3D model, resulting in a 9.02 Å map. This model was low-pass filtered to 60 Å and refinement was performed on the full stack of 116,972 particles in Relion 2.1 with C3 symmetry imposed. Map sharpening and post processing in Relion yielded a 4.8 Å structure using the “gold-standard” FSC cutoff of 0.143. The data collection, refinement parameters and model statistics are summarized in Table 2.

**Table 2:** Cryo-EM data collection, refinement parameters and model statistics

Data collection and processing	BG505:BF520.1 complex (EMDB-9166) (PDB 6MN7)
Magnification	130,000
Voltage (kV)	300
Electron exposure (e–/Å^2^)	66
Defocus range (μm)	1.0–3.5
Pixel size (Å)	0.55
Symmetry imposed	C3
Initial particle images (no.)	559,022
Final particle images (no.)	118,972
Map resolution (Å)	4.8
FSC threshold	0.143
Map resolution range (Å)	4.53–7.52
Refinement	
Initial model used (PDB code)	–
Model resolution (Å)	9.02
FSC threshold	0.143
Model resolution range (Å)	9.02
Map sharpening *B* factor (Å^2^)	−179.793
Model composition	
Non-hydrogen atoms	21,447
Protein residues	2595
Ligands	192
R.m.s. deviations	
Bond lengths (Å)	0.85
Bond angles (°)	0.96
Validation	
Clashscore	35
Poor rotamers (%)	1
Ramachandran plot	
Favored (%)	89
Allowed (%)	9
Disallowed (%)	2

### Model building

The atomic model was generated by first fitting the BG505.SOSIP.664 trimer from the BG505:PGT128 cryo-EM structure (PDB ID: 5ACO)^34^, with glycans temporarily removed, into the generated 4.8 Å map using the fitmap command in UCSF Chimera^65^. Overall, docking of the BG505 structure showed a good quality fit resulting in a correlation score of 0.8602. No further modification to the protein structure was performed. Both glycans at positions N332 and N301 were manually placed into their corresponding densities in the 4.8 Å map.

The BF520.1 variable heavy and light chains were submitted as a single-polypeptide sequence to MUlti-Sources ThreadER (MUSTER) Online to predict the structure of BF520.1^66^. The structure template used for structure determination was a single-chain variant of anti-gp120 antibody, b12 (PDB ID:3JUY)^67^. Using Chimera’s fitmap command, the BG505.SOSIP.664 trimer and BF520.1 variable domain were sequentially docked into the 4.8 Å map.

## Supplementary information

Supplementary Information

Supplementary Dataset 1

Reporting summary

## Data Availability

The datasets generated and analyzed during the current study are publicly available: BF520 antibody sequencing data (Fig. 1, SRA accession PRJNA510130 [https://www.ncbi.nlm.nih.gov/bioproject/PRJNA510130]) and cryo-EM structure (Fig. 5, Supplementary Fig. 5, PDB accession 6MN7 [https://www.rcsb.org/structure/6mn7]). Antibody variable region sequences for the inferred BF520.1 lineage intermediates, inferred naives, and minimal mutants are available in Supplementary Dataset 1.

## Code availability

Custom code is publicly available: prune.py, https://git.io/fh94Y and ecgtheow, https://github.com/matsengrp/ecgtheow.
