# Supplementary material for: Kappa chain maturation helps drive rapid development of an infant HIV-1 broadly neutralizing antibody lineage
Source: Nat Commun. 2019 May 16;10:2190. doi: 10.1038/s41467-019-09481-7 (PMC6522554; doi:10.1038/s41467-019-09481-7)
Supplement: Supplementary file 1 — Supplementary Information [file 41467_2019_9481_MOESM1_ESM.pdf]

Supplementary Information for

Kappa chain maturation helps drive rapid development of an infant HIV-1 broadly neutralizing antibody lineage

Cassandra A Simonich[1,2†], Laura Doepker[1†], Duncan Ralph[3], James A Williams[4], Amrit Dhar[3,5], Zak Yaffe[2], Lauren Gentles[6,7], Christopher T Small[3], Brian Oliver[8], Vladimir Vigdorovich[8], Vidya Mangala Prasad[4], Ruth Nduati[9], D Noah Sather[8], Kelly K Lee[4], Frederick A Matsen IV[3], Julie Overbaugh[1,2*]

[1]Division of Human Biology, Fred Hutchinson Cancer Research Center, Seattle, WA 98109, USA
[2]Medical Scientist Training Program, University of Washington School of Medicine, Seattle, WA 98195, USA
[3]Public Health Sciences Division, Fred Hutchinson Cancer Research Center, Seattle, WA 98109, USA
[4]Department of Medicinal Chemistry, University of Washington, Seattle, WA 98195, USA
[5]Department of Statistics, University of Washington, Seattle, WA 98195, USA
[6]Department of Microbiology, University of Washington, Seattle, WA 98195, USA
[7]Division of Basic Sciences, Fred Hutchinson Cancer Research Center, Seattle, WA 98109, USA
[8]Center for Infectious Disease Research, Seattle, WA 98109, USA
[9]Department of Pediatrics and Child Health, University of Nairobi, Nairobi, Kenya
*Corresponding author and lead contact: joverbau@fredhutch.org
†These authors contributed equally

Nature Communications, 2019

Supplementary Table 1

BF520 time point	Live PBMC count	PBMC viability	Replicate	Chain	Raw reads	Replicate-merged deduplicated sequences	BF520.1 lineage reads	Lineage overlap by replicates	BF520.1 lineage reads	Singlets	Doublets	Triplets	Multiplets (one sequence represented by each multiplicity value)
M9 (M6 pi)	7170000	93.14%	1	IgG	606016	355208	1126	0	584	576	7	1	0
			2	IgG	416253				542	541	1	0	0
			1	IgK	640947	823671	5174	0	2182	2058	99	19	4, 4, 5, 15, 26, 28
			2	IgK	1090670				2992	2902	70	15	4, 4, 4, 5, 10

Supplementary Table 1. BF520 next-generation sequencing results and BF520.1 mAb lineage summary.

Supplementary Table 2

%	IGHV	%	IGKV	%	IGLV
46.72	4-34*01	35.88	4-1*01	15.66	2-14*01+T165G
10.87	3-9*03	14.31	3-15*01	11.20	2-23*03
6.64	3-21*01	6.60	1-5*03	9.99	6-57*01
6.22	3-23*01	5.90	2-28*01	7.99	2-14*01+G156T
4.97	1-69*13	5.35	1-39*01	6.62	1-40*01
2.87	3-7*01	4.95	2D-29*02	5.10	2-11*01
2.38	4-39*01	4.79	3-11*01	5.00	2-8*01
2.31	4-38-2*01	4.55	3-20*01	4.48	8-61*01
1.63	1-2*02+G36A	2.88	2-30*02	4.37	4-69*01
1.36	3-74*01	2.13	1-12*01	4.14	3-25*02
1.31	5-51*01	1.96	1-33*01	3.63	3-10*01
1.21	2-5*01	1.42	1-9*01	3.40	9-49*01
1.02	3-33*01	1.22	1-NL1*01	3.12	1-47*01
0.98	7-4-1*02	1.15	6-21*01	2.59	1-44*01
0.94	1-18*01	1.00	1-13*02	2.34	3-21*03
0.94	1-2*02	0.75	3-15*01+T271C.A272G.A275G	2.08	3-1*01
0.82	1-46*01	0.72	3-11*01+C232A.A234G.G235C.C238T	1.51	7-46*01
0.81	1-24*01	0.54	1-8*01	1.33	1-51*01
0.68	4-59*08	0.54	1-NL1*01+T271C.A272T	1.04	5-45*01
0.58	1-8*02	0.44	1-27*01	0.94	7-43*01
0.57	3-48*01	0.41	1-6*01	0.80	3-27*01
0.53	3-15*01	0.36	1D-16*01	0.75	3-19*01
0.49	3-48*03	0.35	1-9*01+A222C.A243T.T264C	0.52	4-60*03
0.37	4-38-2*02	0.31	2D-29*02+A286G.G287C.A289C	0.40	10-54*01
0.33	3-30*07	0.28	1-17*03	0.23	2-14*01
0.33	1-69-2*01	0.27	1-16*02	0.21	1-36*01
0.32	3-53*01	0.27	5-2*01	0.13	5-39*01
0.30	3-49*04	0.19	2D-26*01	0.13	5-37*01
0.27	3-11*04	0.19	1-NL1*01+T264C	0.12	3-9*01
0.18	1-3*01	0.14	1D-8*01	0.10	2-18*02
0.18	2-70*10	0.13	1-17*01	0.07	5-52*01
0.17	3-72*01	-	-	-	-
0.16	4-31*03	-	-	-	-
0.14	1-58*02	-	-	-	-
0.13	4-4*07	-	-	-	-
0.09	4-4*02	-	-	-	-
0.09	3-20*01	-	-	-	-
0.07	3-64*01	-	-	-	-
%	IGHD	%	IGKD	%	IGLD
13.76	6-13*01	-	-	-	-
10.99	4-17*01	-	-	-	-
10.96	2-2*02	-	-	-	-
10.75	3-3*02	-	-	-	-
6.97	6-6*01	-	-	-	-
6.43	3-3*01	-	-	-	-
6.28	2-2*01	-	-	-	-
6.03	3-10*01	-	-	-	-
4.39	3-10*02	-	-	-	-
3.66	1-26*01	-	-	-	-
3.12	3-22*01	-	-	-	-
2.75	6-19*01	-	-	-	-
2.24	5-18*01	-	-	-	-
1.85	1-1*01	-	-	-	-
1.73	5-12*01	-	-	-	-
1.62	7-27*01	-	-	-	-
1.30	2-15*01	-	-	-	-
1.20	3-16*02	-	-	-	-
0.90	2-21*02	-	-	-	-
0.88	1-7*01	-	-	-	-
0.56	3-9*01	-	-	-	-
0.39	6-25*01	-	-	-	-
0.33	2-8*01	-	-	-	-
0.32	2-8*02	-	-	-	-
0.31	3-16*01	-	-	-	-
0.28	1-20*01	-	-	-	-
%	IGHJ	%	IGKJ	%	IGLJ
72.86	4*02	34.72	1*01	55.94	3*02
11.47	1*01	24.26	2*01	39.20	2*01
6.27	3*02	23.17	4*01	3.84	1*01
3.56	5*02	10.70	3*01	1.03	7*01
2.40	2*01	7.15	5*01	-	-
2.37	6*02	-	-	-	-
1.07	6*03	-	-	-	-

Supplementary Table 2. BF520 inferred Ig germline genes. Shown with percent prevalence for IgM, IgK, and IgL. Newly inferred alleles are indicated by a plus sign, and the point mutations separating each from its nearest known counterpart. This inference was performed only for V, and not for D or J genes. Alleles corresponding to the BF520.1 antibody are highlighted in red.

Supplementary Fig. 1

a

**BEAST (N=758)**	ASR Probability < 0.8	ASR Probability >= 0.8	P(high-confidence ASR sequences on True Lineage)
Is ASR sequence off True Lineage?	0.89	0.0026	0.97
Is ASR sequence on True Lineage?	0.0092	0.095	
**DNAML (N=90)**	ASR Probability < 0.8	ASR Probability >= 0.8	P(high-confidence ASR sequences on True Lineage)
Is ASR sequence off True Lineage?	0	0.12	0.87
Is ASR sequence on True Lineage?	0.044	0.83	
**DNAPARS (N=88)**	ASR Probability < 0.8	ASR Probability >= 0.8	P(high-confidence ASR sequences on True Lineage)
Is ASR sequence off True Lineage?	0	0.1	0.89
Is ASR sequence on True Lineage?	0.057	0.84	
**RevBayes (N=1052)**	ASR Probability < 0.8	ASR Probability >= 0.8	P(high-confidence ASR sequences on True Lineage)
Is ASR sequence off True Lineage?	0.92	0.0029	0.96
Is ASR sequence on True Lineage?	0.0076	0.067	

b

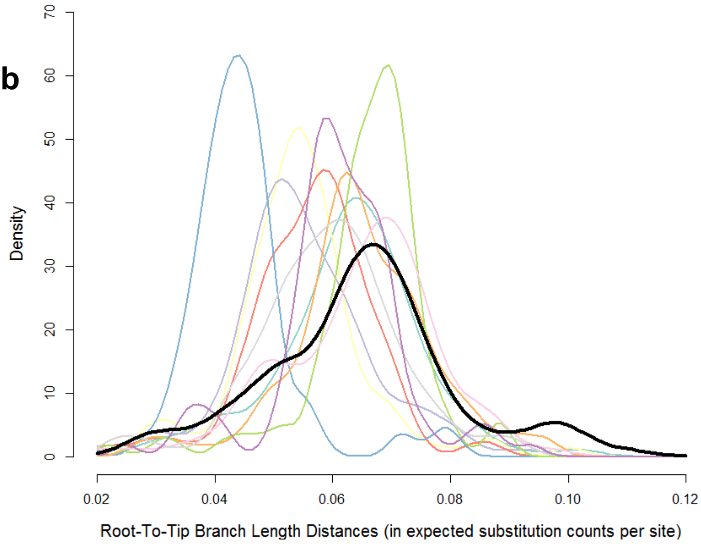

Supplementary Fig. 1: Accuracy of Bayesian lineage reconstruction for BF520.1-like simulated sequence alignments. a, Comparison of BEAST, dnaml, dnapars, and RevBayes methods for antibody lineage reconstruction based on simulated antibody phylogenies. True Lineages were defined as the naïve-to-seed lineages on simulated clonal family trees. **b**, Comparison of the root-to-tip branch length distance distribution for the BF520.1 clonal family (in black) against the corresponding distributions for 10 independently simulated clonal families (in lighter colors), showing concordance between the simulations and the observed distribution. To compute the distribution for the BF520.1 family, we obtained 10,000 independent tree samples from RevBayes and computed the median root-to-tip branch length distance for each sequence in the clonal family. Each of the 10 simulated clonal families were obtained using the same simulation parameters. ASR: ancestral sequence reconstruction.

Supplementary Fig. 2

a

BF520.1 lineage heavy chain intermediates

Tier	Clade	VH / VK	naïveVH / naïveVK	naïveVH / matureVK	Int1VH / matureVK	Int2VH / matureVK	Int3VH / matureVK	Int4VH / matureVK	Int5VH / matureVK	Int6VH / matureVK	matureVH / matureVK
1A	B	SF162	>20	0.7	0.6	<0.6	<0.6	<0.6	<0.6	<0.6	<0.6
1B	A	Q23.17	>20	1.1	2.2	0.9	<0.6	<0.6	<0.6	<0.6	<0.6
2	A	BG505.W6M.C2_T332N	>20	>20	>20	7.0	3.1	1.7	1.0	1.3	1.1
2	A	398F1	>20	5.5	4.9	3.7	1.3	0.6	<0.6	<0.6	<0.6
2	B	X2278	>20	19.5	>20	3.8	2.3	1.2	0.7	0.9	1.0
2	B	TRO.11	>20	>20	>20	>20	>20	6.5	4.8	7.9	5.9
2	BC	BJOX002000	>20	>20	>20	15.8	3.4	1.0	0.8	1.3	0.9
2	BC	CH119	>20	>20	>20	>20	14.7	6.1	3.7	6.1	6.0
2	C	CE1176	>20	17.0	17.9	13.5	8.7	6.1	5.6	7.0	5.1
2	C	QC406.F3	>20	<0.6	0.7	<0.6	<0.6	<0.6	<0.6	<0.6	<0.6
2	C	CE0217	>20	>20	>20	>20	12.3	2.1	2.1	4.4	2.8
2	C	DU156.12	>20	>20	>20	16.9	9.8	2.0	1.8	2.3	2.2
2	C	DU422.1	>20	5.7	12.9	3.2	4.4	2.2	1.4	2.9	2.6
		SIV	>20	>20	>20	>20	>20	>20	>20	>20	>20

b

BF520.1 VH maximum likelihood lineage intermediates

Tier	Clade	VH / VK	naïveVH / mature VK	ML Int1VH / mature VK	ML Int2VH / mature VK	ML Int3VH / mature VK	ML Int4VH / mature VK	ML Int5VH / mature VK	matureVH / mature VK
1A	B	SF162	0.7	0.7	<0.6	<0.6	<0.6	<0.6	<0.6
1B	A	Q23.17	1.1	2.0	3.6	1.2	<0.6	<0.6	<0.6
2	A	BG505.W6M.C2_T332	>20	>20	>20	>20	6.3	2.5	1.1
2	A	398F1	5.5	>20	>20	19.6	2.4	1.3	<0.6
2	B	X2278	19.5	18.8	19.9	7.1	2.8	1.3	1.0
2	B	TRO.11	>20	>20	>20	>20	>20	15.3	5.9
2	BC	BJOX002000	>20	>20	>20	>20	11.9	2.8	0.9
2	BC	CH119	>20	>20	>20	>20	>20	8.8	6.0
2	C	CE1176	17.0	>20	>20	>20	8.3	7.0	5.1
2	C	QC406.F3	<0.6	<0.6	1.3	0.8	<0.6	<0.6	<0.6
2	C	CE0217	>20	>20	>20	>20	14.6	6.0	2.8

c

BF520.1 heavy chain (maximum likelihood)

FR1 | CDRH1 | FR2 | CDRH2 | FR3 | CDRH3 | FR4

```
               10        20        30        40        50        60        70        80        90        100       110       120
Naive QVQLVQSGAEVKKPGASVKVSCKASGYTFTGYYMHWVRQAPGQGLEWMGWINPNSGGTNYAQKFQGRVTMTRDTSISTAYMELSRLRSDDTAVYYCARGPFPNYYGPGSYWGGFDYWGQGTLVTVSS
Int1  ----------------------------------------------------------------------------------------------------------S--------------------
Int2  --------------------------------------------------------D--------A-----------------N--K-------------------S--------------------
Int3  -------------------------------N------------------------D--------A-----------------N--K-------------------S--------------------
Int4  -------------------------------N------------------------D----------------------------------------------------------------------
Int5  -------------------------------N-------------------A----D---------------------------------------------------------------------- >M6pi
VH    ----------M-M------------------N-I-----------------A-H--D-S---R--------G---L-------------------------------------L-F-------S---
```

d

BF520.1 VH parsimony lineage intermediates

Tier	Clade	VH / VK	naïve VH / matureVK	Pars Int1 VH / matureVK	Pars Int2 VH / matureVK	Pars Int3 VH / matureVK	Pars Int4 VH / matureVK	Pars Int5 VH* / matureVK	mature VH / matureVK
1A	B	SF162	0.7	<0.6	<0.6	<0.6	<0.6	<0.6	<0.6
1B	A	Q23.17	1.1	0.9	1.4	1.2	<0.6	<0.6	<0.6
2	A	BG505.W6M.C2_T332N	>20	>20	>20	>20	3.0	1.7	1.1
2	A	398F1	5.5	5.2	15.7	11.7	1.4	0.6	<0.6
2	B	X2278	19.5	14.1	19.7	15.8	1.7	1.2	1.0
2	B	TRO.11	>20	>20	>20	>20	15.1	6.5	5.9
2	BC	BJOX002000	>20	>20	>20	>20	3.1	1.0	0.9
2	BC	CH119	>20	>20	>20	>20	10.6	6.1	6.0
2	C	CE1176	17.0	17.9	>20	>20	6.3	6.1	5.1
2	C	QC406.F3	<0.6	<0.6	0.7	0.9	<0.6	<0.6	<0.6
2	C	CE0217	>20	>20	>20	>20	11.6	2.1	2.8

* same mAb as the Bayesian lineage VH Int4

e

BF520.1 heavy chain (parsimony)

FR1 | CDRH1 | FR2 | CDRH2 | FR3 | CDRH3 | FR4

```
               10        20        30        40        50        60        70        80        90        100       110       120
Naïve QVQLVQSGAEVKKPGASVKVSCKASGYTFTGYYMHWVRQAPGQGLEWMGWINPNSGGTNYAQKFQGRVTMTRDTSISTAYMELSRLRSDDTAVYYCARGPFPNYYGPGSYWGGFDYWGQGTLVTVSS
Int1  -------------------------------------------------------------------------------------------------------------------------------
Int2  --------------------------------------------------------D----------------------------------------------------------------------
Int3  ---------------------------------I----------------------D----------------------------------------------------------------------
Int4  -------------------------------N-I----------------------D--------------------------------------------------------L-------------
Int5  -------------------------------N-I-----------------A----D--------------------------------------------------------L------------- >M6pi
VH    ----------M-M------------------N-I-----------------A-H--D-S---R--------G---L-------------------------------------L-F-------S---
```

Supplementary Fig. 2: Development of heterologous neutralization by the maturing BF520.1 heavy chain lineage. **a**,**b**,**d**, mAb neutralization of panel viruses by BF520.1 inferred Bayesian (**a**), Maximum likelihood (**b**), and Parsimony (**d**) lineage heavy chains paired with the mature kappa light chain. The top rows of the tables (VH and VK) show the origin of the antibody chain sequence used indicated by $Int\#_{VH}$ with # indicating the progression of intermediates in the lineage. These were paired with the indicated kappa chain, in most cases the mature kappa chain ($mature_{VK}$). Panel viruses are shown to the left, with the tier, clade and name indicated. IC_{50} values (μg ml^{-1}) represent an average of two to three independent experiments performed in duplicate. IC_{50} values are color-coded with darker shades indicating more potent neutralization. Grey indicates that 50% neutralization was not achieved at the highest mAb concentration tested. **c**,**e**, Amino acid alignments of maximum likelihood (**c**) and parsimony (**e**) heavy chain lineage intermediates.

Supplementary Fig. 3: Development of heterologous neutralization by the maturing BF520.1 kappa light chain lineage. **a**,**b**,**d**, mAb neutralization of panel viruses by BF520.1 inferred Bayesian (**a**), Maximum likelihood (**b**), and Parsimony (**d**) lineage kappa light chains paired with the mature heavy chain. Layout and details are as described for Supplementary Fig. 2. **c**,**e**, Amino acid alignments of maximum likelihood (**c**) and parsimony (**e**) kappa light chain lineage intermediates.

Supplementary Fig. 3

a

BF520.1 lineage kappa chain intermediates

Tier	Clade	VH / VK	naïve$_{VH}$ / naïve$_{VK}$	mature$_{VH}$ / naïve$_{VK}$	mature$_{VH}$ / Int1$_{VK}$	mature$_{VH}$ / Int2$_{VK}$	mature$_{VH}$ / Int3$_{VK}$	mature$_{VH}$ / Int4$_{VK}$	mature$_{VH}$ / Int5$_{VK}$	mature$_{VH}$ / Int6$_{VK}$	mature$_{VH}$ / Int7$_{VK}$	mature$_{VH}$ / mature$_{VK}$
1A	B	SF162	>20	1.1	0.9	<0.6	<0.6	<0.6	<0.6	<0.6	<0.6	<0.6
1B	A	Q23.17	>20	>20	>20	1.0	<0.6	0.7	0.7	<0.6	<0.6	<0.6
2		BG505.W6M.C2_T332N	>20	>20	>20	18.7	3.2	2.2	1.6	1.0	1.3	1.1
		398F1	>20	>20	>20	10.3	1.7	1.5	1.0	0.7	<0.6	<0.6
	B	X2278	>20	>20	>20	11.7	2.5	1.4	1.1	0.9	0.9	1.0
		TRO.11	>20	>20	>20	>20	>20	>20	14.6	7.0	8.0	5.9
	BC	BJOX002000	>20	>20	>20	>20	5.7	3.5	1.6	0.8	1.1	0.9
		CH119	>20	>20	>20	>20	>20	18.3	8.8	5.2	4.5	6.0
	C	CE1176	>20	>20	>20	15.0	8.2	6.9	6.3	5.3	5.6	5.1
		QC406.F3	>20	>20	>20	1.1	<0.6	<0.6	<0.6	<0.6	<0.6	<0.6
		CE0217	>20	>20	>20	>20	>20	>20	6.4	3.1	4.7	2.8
		DU156.12	>20	>20	>20	>20	>20	15.7	3.7	0.9	2.1	2.2
		DU422.1	>20	>20	>20	11.0	5.1	5.4	5.2	3.9	4.3	2.6
		SIV	>20	>20	>20	>20	>20	>20	>20	>20	>20	>20

b

BF520.1 VK maximum likelihood lineage intermediates

Tier	Clade	VH / VK	mature$_{VH}$ / naïve$_{VK}$	ML Int1$_{VK}$*	ML Int2$_{VK}$**	ML Int3$_{VK}$	ML Int4$_{VK}$	ML Int5$_{VK}$***	mature$_{VK}$
1A	B	SF162	1.1	<0.6	<0.6	<0.6	<0.6	<0.6	<0.6
1B	A	Q23.17	>20	>20	4.8	1.7	<0.6	0.7	<0.6
2		BG505.W6M.C2_T332N	>20	>20	>20	>20	2.0	2.2	1.1
		398F1	>20	>20	>20	>20	1.4	2.4	<0.6
	B	X2278	>20	>20	>20	>20	2.8	2.9	1.0
		TRO.11	>20	>20	>20	>20	>20	>20	5.9
	BC	BJOX002000	>20	>20	>20	>20	5.3	5.5	0.9
		CH119	>20	>20	>20	>20	12.9	>20	6.0
	C	CE1176	>20	>20	>20	>20	6.2	7.3	5.1
		QC406.F3	>20	19.7	17.2	7.5	<0.6	<0.6	<0.6
		CE0217	>20	>20	>20	>20	>20	>20	2.8

* same mAb as Pars Int1$_{VK}$
** same mAb as Pars Int2$_{VK}$
*** same mAb as Pars Int6$_{VK}$

c

BF520.1 light chain (maximum likelihood)

```
                FR1             CDRL1        FR2       CDRL2            FR3                    CDRL3       FR4
               10         20         30         40         50         60         70         80         90        100
Naïve EIVMTQSPATLSVSPGERATLSCRASQSVSSNLAWYQQKPGQAPRLLIYGASTRATGIPARFSGSGSGTEFTLTISSLQSEDFAVYYCQQYNNWPSPFTFGPGTKVDIK
Int1  -----------------------------------------------------------------------------M-------------------------------
Int2  ------------------------------------------------------------------F----------M-------------------------------
Int3  ----------------------------------------------------S-------------F----------M------------------------------E
Int4  ---------------------------N-A----------------------S-------------F----------M------------------------------E
Int5  ---------------------------N-A----------------------S-------------F----------M-------------------------------
VK    --------------L---------T--N-AY-F----------------E--S----T--------F----------M----------------------------H--
```

d

BF520.1 VK parsimony lineage intermediates

Tier	Clade	VH / VK	mature$_{VH}$ / naïve$_{VK}$	Pars Int1$_{VK}$*	Pars Int2$_{VK}$**	Pars Int3$_{VK}$	Pars Int4$_{VK}$	Pars Int5$_{VK}$	Pars Int6$_{VK}$***	Pars Int7$_{VK}$	Pars Int8$_{VK}$	mature$_{VK}$
1A	B	SF162	1.1	<0.6	<0.6	<0.6	<0.6	<0.6	<0.6	<0.6	<0.6	<0.6
1B	A	Q23.17	>20	>20	4.8	2.3	17.6	<0.6	0.7	<0.6	<0.6	<0.6
2		BG505.W6M.C2_T332N	>20	>20	>20	>20	>20	1.7	2.2	2.4	0.7	1.1
		398F1	>20	>20	>20	>20	>20	2.1	2.4	1.9	0.8	<0.6
	B	X2278	>20	>20	>20	>20	>20	2.4	2.9	2.2	1.2	1.0
		TRO.11	>20	>20	>20	>20	>20	>20	>20	>20	16.0	5.9
	BC	BJOX002000	>20	>20	>20	>20	>20	3.7	5.5	2.8	0.8	0.9
		CH119	>20	>20	>20	>20	>20	>20	>20	12.6	4.1	6.0
	C	CE1176	>20	>20	>20	>20	>20	6.7	7.3	6.4	4.2	5.2
		QC406.F3	>20	19.7	17.2	8.0	18.5	<0.6	<0.6	<0.6	<0.6	<0.6
		CE0217	>20	>20	>20	>20	>20	>20	>20	>20	6.9	2.8

* same mAb as ML Int1$_{VK}$
** same mAb as ML Int2$_{VK}$
*** same mAb as ML Int5$_{VK}$

e

BF520.1 light chain (parsimony)

```
                FR1             CDRL1        FR2       CDRL2            FR3                    CDRL3       FR4
               10         20         30         40         50         60         70         80         90        100
Naïve EIVMTQSPATLSVSPGERATLSCRASQSVSSNLAWYQQKPGQAPRLLIYGASTRATGIPARFSGSGSGTEFTLTISSLQSEDFAVYYCQQYNNWPSPFTFGPGTKVDIK
Int1  -----------------------------------------------------------------------------M-------------------------------
Int2  ------------------------------------------------------------------F----------M-------------------------------
Int3  ----------------------------------------------------N---A---------F----------M-------------------------------
Int4  -----------------------------T----------------------N---A---------F----------M-------------------------------
Int5  ---------------------------N-A----------------------N---A---------F----------M-------------------------------
Int6  ---------------------------N-A----------------------S-------------F----------M-------------------------------
Int7  ---------------------------N-AY---------------------S-------------F----------M-------------------------------
                                                                                                                 >M6pi
Int8  ------------------------T--N-AY-F-------------------S-------------F----------M-------------------------------
VK    --------------L---------T--N-AY-F----------------E--S----T--------F----------M----------------------------H--
```

Supplementary Fig. 4

a

BF520.1 lineage paired intermediates

Tier	Clade		naive	Int1	Int2	Int3	Int3	Int4	Int4	Int4	Int5	Int6	mature	Minimal
		VH	naive$_{VH}$	Int1$_{VH}$	Int2$_{VH}$	Int3$_{VH}$	Int3$_{VH}$	Int4$_{VH}$	Int4$_{VH}$	Int4$_{VH}$	Int5$_{VH}$	Int6$_{VH}$	mature$_{VH}$	Minimal$_{VH}$
		% SHM		0.5	0.8	1.3	1.3	1.8	1.8	1.8	2.4	2.6	6.5	1.3
		VK	naive$_{VK}$	Int1$_{VK}$	Int2$_{VK}$	Int3$_{VK}$	Int4$_{VK}$	Int4$_{VK}$	Int5$_{VK}$	Int6$_{VK}$	Int6$_{VK}$	Int7$_{VK}$	mature$_{VK}$	Minimal$_{VK}$
Tier	Clade	% SHM		0.3	1.2	1.8	2.1	2.1	3.4	3.7	3.7	4.3	4.6	2.7
1A	B	SF162	>20	>20	1.7	<0.6	<0.6	<0.6	<0.6	<0.6	<0.6	<0.6	<0.6	<0.6
1B	A	Q23.17	>20	>20	>20	2.1	1.2	<0.6	<0.6	<0.6	<0.6	0.7	<0.6	<0.6
2		BG505.W6M.C2_T332N	>20	>20	>20	>20	>20	6.2	2.1	1.4	1.0	1.6	1.1	1.3
		398F1	>20	>20	>20	>20	>20	2.0	1.2	<0.6	<0.6	0.8	<0.6	0.7
	B	X2278	>20	>20	>20	>20	>20	2.2	1.3	0.7	0.7	0.9	1.0	1.7
		TRO.11	>20	>20	>20	>20	>20	>20	18.9	10.3	6.0	6.7	5.9	14.2
	BC	BJOX002000	>20	>20	>20	>20	>20	11.9	1.6	0.8	0.7	0.9	0.9	2.5
		CH119	>20	>20	>20	>20	>20	>20	8.2	4.4	3.0	5.7	6.0	16.0
	C	CE1176	>20	>20	>20	>20	>20	7.5	3.7	4.0	4.1	7.4	5.1	5.7
		QC406.F3	>20	>20	>20	3.1	1.1	<0.6	<0.6	<0.6	<0.6	<0.6	<0.6	<0.6
		CE0217	>20	>20	>20	>20	>20	>20	15.0	5.4	3.7	4.9	2.8	5.6
		DU156.12	>20	>20	>20	>20	>20	>20	4.1	2.2	2.0	2.2	2.2	14.8
		DU422.1	>20	>20	>20	10.3	8.2	2.8	2.9	2.3	1.5	4.1	2.6	2.6
		SIV	>20	>20	>20	>20	>20	>20	>20	>20	>20	>20	>20	>20

b

BF520.1 maximum likelihood lineage

Tier	Clade		ML Int1	ML Int3	ML Int4	ML Int5	mature
		VH	ML Int1$_{VH}$	ML Int3$_{VH}$	ML Int4$_{VH}$	ML Int5$_{VH}$	mature$_{VH}$
		%SHM	1.0	2.1	0.8	1.3	6.5
		VK	ML Int1$_{VK}$	ML Int3$_{VK}$	ML Int4$_{VK}$	ML Int5$_{VK}$	mature$_{VK}$
Tier	Clade	%SHM	0.3	1.5	2.4	1.8	4.6
1A	B	SF162	>20	15.9	<0.6	<0.6	<0.6
1B	A	Q23.17	>20	>20	7.0	0.9	<0.6
2		BG505.W6M.C2_T332N	>20	>20	>20	>20	1.1
		398F1	>20	>20	>20	13.7	<0.6
	B	X2278	>20	>20	>20	>20	1.0
		TRO.11	>20	>20	>20	>20	5.9
	BC	BJOX002000	>20	>20	>20	>20	0.9
		CH119	>20	>20	>20	>20	6.0
	C	CE1176	>20	>20	>20	17.5	5.1
		QC406.F3	>20	>20	7.4	1.8	<0.6
		CE0217	>20	>20	>20	>20	2.8

c

BF520.1 parsimony lineage

Tier	Clade		Pars Int1	Pars Int3	Pars Int4	Pars Int4	Pars Int4	Pars Int5	Pars Int5	Pars Int5	mature
		VH	Pars Int1$_{VH}$	Pars Int3$_{VH}$	Pars Int4$_{VH}$	Pars Int4$_{VH}$	Pars Int4$_{VH}$	Pars Int5$_{VH}$	Pars Int5$_{VH}$	Pars Int5$_{VH}$	mature$_{VH}$
		%SHM	0.5	0.8	1.3	1.3	1.3	1.8	1.8	1.8	6.5
		VK	Pars Int1$_{VK}$	Pars Int3$_{VK}$	Pars Int4$_{VK}$	Pars Int5$_{VK}$	Pars Int6$_{VK}$	Pars Int6$_{VK}$	Pars Int7$_{VK}$	Pars Int8$_{VK}$	mature$_{VK}$
Tier	Clade	%SHM	0.3	1.2	1.5	2.1	1.8	1.8	2.4	3.0	4.6
1A	B	SF162	>20	>20	13.9	<0.6	<0.6	<0.6	<0.6	<0.6	<0.6
1B	A	Q23.17	>20	>20	>20	2.5	3.8	0.9	0.7	<0.6	<0.6
2		BG505.W6M.C2_T332N	>20	>20	>20	>20	>20	13.1	6.8	1.5	1.1
		398F1	>20	>20	>20	>20	19.6	2.6	2.1	0.8	<0.6
	B	X2278	>20	>20	>20	>20	>20	8.9	3.7	1.2	1.0
		TRO.11	>20	>20	>20	>20	>20	>20	>20	>20	5.9
	BC	BJOX002000	>20	>20	>20	>20	20.0	15.8	13.1	1.5	0.9
		CH119	>20	>20	>20	>20	>20	>20	>20	5.1	6.0
	C	CE1176	>20	>20	>20	>20	>20	11.4	9.6	4.3	5.1
		QC406.F3	>20	>20	>20	3.0	4.4	<0.6	<0.6	<0.6	<0.6
		CE0217	>20	>20	>20	>20	>20	>20	>20	9.0	2.8

Supplementary Fig. 4: Neutralization properties of paired VH and VK lineage intermediates. a,**b**,**c**, Neutralization of panel viruses by BF520.1 Bayesian (**a**), maximum likelihood (**b**) and parsimony (**c**) lineage heavy and light chain paired intermediates. IC_{50} values in μg ml^{-1}. The top rows indicate the pairings of lineage intermediates for both VH and VK chains. Below each the % SHM in nucleotides is indicated. Layout and details are as described for Supplementary Fig. 2.

Supplementary Fig. 5

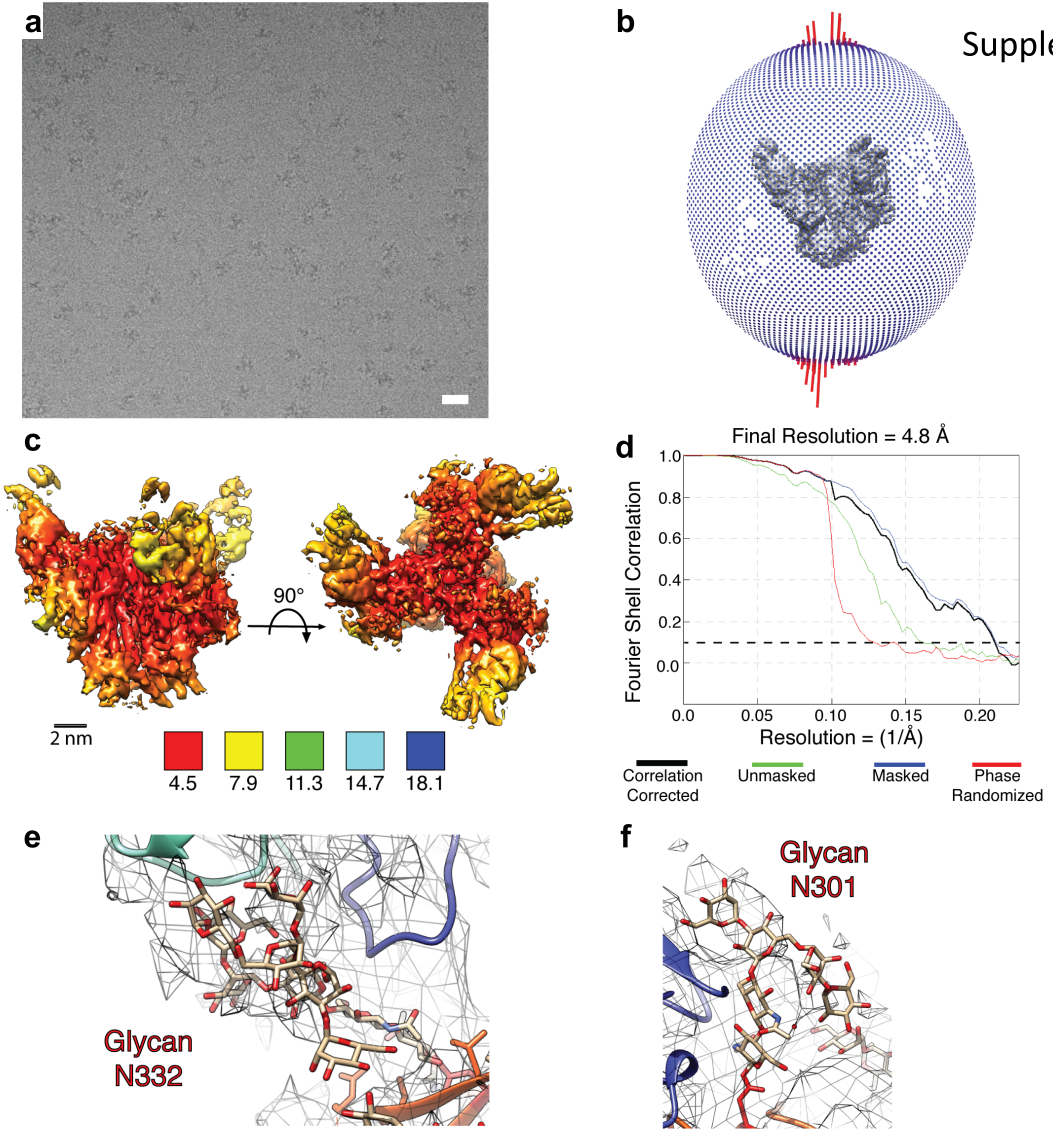

g

Fit	Molecule	Correlation
1	**BF520.1**	**0.8513**
2	BF520.1	0.6995
3	BF520.1	0.6991
4	BF520.1	0.6732

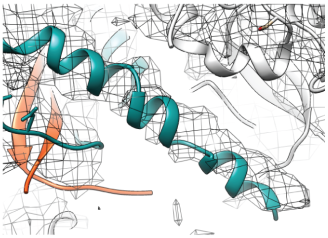

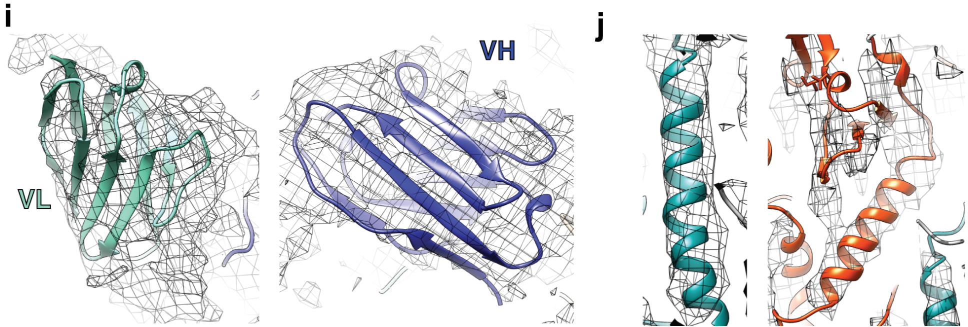

Supplementary Fig. 5: Data collection and refinement of BG505.SOSIP.664 in complex with BF520.1 Fab. a, A sample, frame aligned, micrograph illustrating particle density and distribution. **b**, Angular distribution plot generated in Relion/2.1. **c**, Local resolution analysis of the 4.8 Å cryo-EM reconstruction. The color key shown below indicates local resolution in Å. **d**, Fourier-shell correlation curves for correlation corrected (black), unmasked (green), masked (blue) and phase randomized (red). Dashed line represents the “gold-standard” FSC cutoff of 0.143. **e**,**f**, Densities corresponding to N332 and N301 glycans and positioning of glycans into the cryo-EM density. **g**, Correlation scores of the top fits for the BF520.1 Fab structure into the cryo-EM map. Scores were generated by Chimera UCSF fitmap command utilizing a global search. **h**, Positioning of gp41 HR2 -helix into the cryo-EM density. **i**, Best fit of BF520.1 homology model determined in (**h**). Shown are the variable light chain (left) and variable heavy chain (right). **j**, Positioning of the g41 HR1 -helix (left) and the gp120 1 helix into the cryo-EM density.

Supplementary Fig. 6

a

```
                FR1                CDR1          FR2        CDR2        FR3
VH_Naive     QVQLVQSGAEVKKPGASVKVSCKASGYTFTGYYMHWVRQAPGQGLEWMGWINPNSGGTNYAQKFQGRVTM
VH_Naive_2   -------------------------------------------------R--------------------

                FR3                      CDR3              FR4
VH_Naive     TRDTSISTAYMELSRLRSDDTAVYYCARGPFPNYYGPGSYWGGFDYWGQGTLVTVSS
VH_Naive_2   ---------------------------------------------------------
```

b

```
                FR1                   CDR1        FR2          CDR2    FR3
VK_Naive     EIVMTQSPATLSVSPGERATLSCRASQSVSSNLAWYQQKPGQAPRLLIYGASTRATGIPARF
VK_Naive_2   --------------------------------------------------------------
VK_Naive_3   --------------------------------------------------------------

                FR3
VK_Naive     SGSGSGTEFTLTISSLQSEDFAVYYCQQYNNWPSPFTFGPGTKVDIK
VK_Naive_2   ---------------M-----------------T-------------
VK_Naive_3   ---------------M-----------------P-------------
```

c

Tier	Clade		VH_Naive VK_Naive	VH_Naive_2 VK_Naive_2	VH_Naive_2 VK_Naive_3
1A	B	SF162	>20	>20	>20
1B	A	Q23.17	>20	>20	>20
2	A	BG505.W6M.C2_T332	>20	>20	>20
		398F1	>20	>20	>20
	B	X2278	>20	>20	>20
		TRO.11	>20	>20	>20
	BC	BJOX002000	>20	>20	>20
		CH119	>20	>20	>20
	C	CE1176	>20	>20	>20
		QC406.F3	>20	>20	>20
		CE0217	>20	>20	>20

Autologous	A	BF520.W14M.E3	>20	>20	>20
		BF520.M6P.D1	>20	>20	>20
		BF520.M6P.H1	>20	>20	>20
		BF520.M6P.I1	>20	>20	>20
		BF520.M6P.J1	>20	>20	>20

d

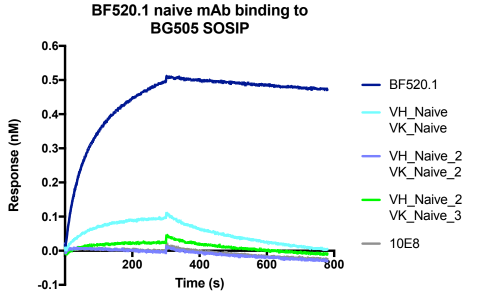

e

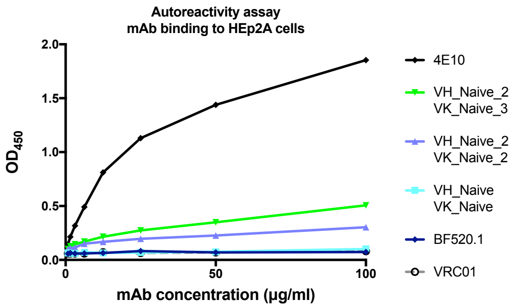

Supplementary Fig. 6: BF520.1 inferred naïve mAbs. a,**b**, Amino acid alignment of heavy chain (**a**) and kappa chain (**b**) inferred naïve sequences. **c**, BF520.1 naïve mAb neutralization of viruses. IC_{50} values (μg ml^{-1}) are an average of two to three independent experiments performed in duplicate. Grey indicates that 50% neutralization was not achieved at the highest mAb concentration tested. **d**, mAb (analyte) binding to the BG505.SOSIP.664 (ligand) measured by BLI. 10E8 is a negative control as the SOSIP trimer does not contain the targeted MPER epitope. Data are representative of two independent experiments. **e**, mAb binding to HEp2A cells measured by ELISA (AESKULISA ANA-HEp-2; Aesku Diagnostics). The MPER-directed bnAb 4E10 is known to be autoreactive and was included as a positive control. VRC01 was included as a negative control. Data are representative of two independent experiments.

Supplementary Fig. 7

a

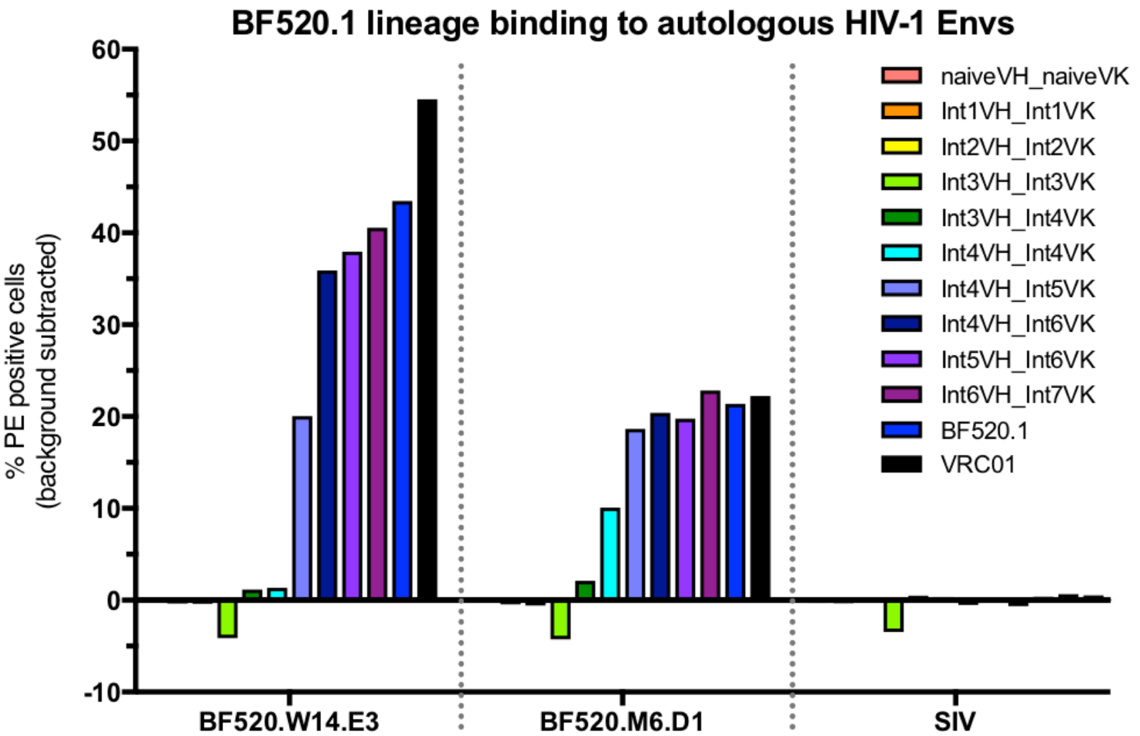

b

BF520.1 VH	naïve_{VH}	Int1_{VH}	Int2_{VH}	Int3_{VH}	Int3_{VH}	Int4_{VH}	Int4_{VH}	Int4_{VH}	Int5_{VH}	Int6_{VH}	mature_{VH}
BF520.1 VK	naïve_{VK}	Int1_{VK}	Int2_{VK}	Int3_{VK}	Int4_{VK}	Int4_{VK}	Int5_{VK}	Int6_{VK}	Int6_{VK}	Int7_{VK}	mature_{VK}
BF520.W14M.E3	>20	>20	>20	>20	>20	>20	>20	>20	>20	>20	>20
BF520.M6P.D1	>20	>20	>20	>20	>20	>20	6.1	0.7	<0.6	<0.6	<0.6
BF520.M6P.H1	>20	>20	>20	>20	>20	>20	>20	11.6	4.6	2.3	1.4
BF520.M6P.I1	>20	>20	>20	>20	>20	5.8	2.1	1.1	0.7	1.6	1.1
BF520.M6P.J1	>20	>20	>20	>20	>20	>20	>20	3.6	1.8	1.7	1.8

Supplementary Fig. 7: Neutralization of autologous virus by BF520.1 paired lineage intermediates. a, mAb binding to cell-surface Env detected by flow cytometry. VRC01 was a positive control for Env expression. The reported values have had background binding to mock-transfected cells subtracted. Data are representative of two independent experiments. **b**, The origin of the VH and VK chains are shown at the top and are described in Fig. 2b and 3d. Viruses tested are shown to the left and include a variant at first HIV detection (14 weeks of age) designated ‘‘W14’’ and four variants from ~2.2 months pi (6 months of age, “M6”). IC_{50} values (μg ml^{-1}) are color coded with darker shading indicating greater neutralization potency. Results are an average of 2 independent experiments.
