# Supplementary material for: Kappa chain maturation helps drive rapid development of an infant HIV-1 broadly neutralizing antibody lineage
Source: Nat Commun. 2019 May 16;10:2190. doi: 10.1038/s41467-019-09481-7 (PMC6522554; doi:10.1038/s41467-019-09481-7)
Supplement: Supplementary file 3 — Reporting summary [file 41467_2019_9481_MOESM3_ESM.pdf]

Corresponding author(s): Julie Overbaugh

Reporting Summary

Nature Research wishes to improve the reproducibility of the work that we publish. This form provides structure for consistency and transparency in reporting. For further information on Nature Research policies, see Authors & Referees and the Editorial Policy Checklist.

Statistical parameters

When statistical analyses are reported, confirm that the following items are present in the relevant location (e.g. figure legend, table legend, main text, or Methods section).

n/a	Confirmed	
☒	☐	The exact sample size (n) for each experimental group/condition, given as a discrete number and unit of measurement
☐	☒	An indication of whether measurements were taken from distinct samples or whether the same sample was measured repeatedly
☒	☐	The statistical test(s) used AND whether they are one- or two-sided<br>*Only common tests should be described solely by name; describe more complex techniques in the Methods section.*
☒	☐	A description of all covariates tested
☒	☐	A description of any assumptions or corrections, such as tests of normality and adjustment for multiple comparisons
☒	☐	A full description of the statistics including central tendency (e.g. means) or other basic estimates (e.g. regression coefficient) AND variation (e.g. standard deviation) or associated estimates of uncertainty (e.g. confidence intervals)
☒	☐	For null hypothesis testing, the test statistic (e.g. F, t, r) with confidence intervals, effect sizes, degrees of freedom and P value noted<br>*Give P values as exact values whenever suitable.*
☐	☒	For Bayesian analysis, information on the choice of priors and Markov chain Monte Carlo settings
☒	☐	For hierarchical and complex designs, identification of the appropriate level for tests and full reporting of outcomes
☒	☐	Estimates of effect sizes (e.g. Cohen's d, Pearson's r), indicating how they were calculated
☒	☐	Clearly defined error bars<br>*State explicitly what error bars represent (e.g. SD, SE, CI)*

Our web collection on statistics for biologists may be useful.

Software and code

Policy information about availability of computer code

Data collection	FLASH v1.2.11, cutadapt 1.14, Python 2.7.9, and FASTX-toolkit 0.0.14 were used for sequence reconstruction and cleanup. EPU Automated Data Collection Software and Leginon v3.2 were used for cryo-EM data collection.
Data analysis	Partis, FastTree 2, Prune.py, PHYLIP v3.696, BEAST v1.8.4, RevBayes, and Ecgtheow were used for sequence analysis. ForteBio's Octet Software Data Analysis 7.0, Prism 7.0c, and FlowJo 10 were used for binding assays. PyMOL, Leginon v3.2, Relion v2.1, Chimera v1.13, MUSTER, MotionCor2 v1.0.4, and CFTFIND4 v4.1.8 were used in cryo-EM data analysis. Unpublished custom code (prune.py and ecgtheow) have been made publicly available via GitHub, links are included in the manuscript.

For manuscripts utilizing custom algorithms or software that are central to the research but not yet described in published literature, software must be made available to editors/reviewers upon request. We strongly encourage code deposition in a community repository (e.g. GitHub). See the Nature Research guidelines for submitting code & software for further information.

Data

Policy information about availability of data

All manuscripts must include a data availability statement. This statement should provide the following information, where applicable:

- Accession codes, unique identifiers, or web links for publicly available datasets
- A list of figures that have associated raw data
- A description of any restrictions on data availability

The datasets generated during and/or analysed during the current study are available from the corresponding author on reasonable request.

Field-specific reporting

Please select the best fit for your research. If you are not sure, read the appropriate sections before making your selection.

☒ Life sciences ☐ Behavioural & social sciences ☐ Ecological, evolutionary & environmental sciences

For a reference copy of the document with all sections, see nature.com/authors/policies/ReportingSummary-flat.pdf

Life sciences study design

All studies must disclose on these points even when the disclosure is negative.

Sample size	Our sample size was one individual because this infant mounted broad and potent antibody responses to HIV.
Data exclusions	No data were excluded from this study.
Replication	We replicated the deep sequencing of our subject's antibody genes. Beyond technical replicates, we replicated all neutralization assays with different stocks of pseudovirus and different individuals performing the assays.
Randomization	We had no randomization in this study of one individual's antibody response.
Blinding	Blinding was not relevant to our study because we had no randomization groups.

Reporting for specific materials, systems and methods

Materials & experimental systems

n/a	Involved in the study	
☐	☒	Unique biological materials
☐	☒	Antibodies
☐	☒	Eukaryotic cell lines
☒	☐	Palaeontology
☒	☐	Animals and other organisms
☐	☒	Human research participants

Methods

n/a	Involved in the study	
☒	☐	ChIP-seq
☒	☐	Flow cytometry
☒	☐	MRI-based neuroimaging

Unique biological materials

Policy information about availability of materials

Obtaining unique materials	The longitudinal blood samples collected from infant BF520 were used in this study and previous studies, so none are available. There are no duplicate samples in existence.

Antibodies

Antibodies used	Human BF520.1 GenBank: KX168065, KX168069. Antibody sequences for the inferred BF520.1 lineage intermediates, inferred naïves, and minimal mutants are available from the corresponding author upon request. As these sequences were inferred, they are not eligible for deposition into GenBank.
Validation	No validation of this antibody lineage was required. Simonich, Cell 2016 describes the discovery of antibody BF520.1.

Eukaryotic cell lines

Policy information about cell lines

Cell line source(s)	293F (Invitrogen R79007), TZM-bl (NIH-ARP 8129)
Authentication	None of these cell lines were authenticated upon receipt from primary sources. We are able to authenticate them if required.
Mycoplasma contamination	All cell lines tested negative for mycoplasma.
Commonly misidentified lines (See ICLAC register)	None used

Human research participants

Policy information about studies involving human research participants

Population characteristics	We studied one individual human participant from the Nairobi Breastfeeding Clinical Trial cohort (Nduati, JAMA, 2000).
Recruitment	This participant was recruited according to the procedures outlined in Nduati, JAMA, 2000. Infant BF520 was in utero during enrollment of the mother-infant pair.

April 2018
